# Supplementary figures and images for: Dynamic changes in brain lateralization correlate with human cognitive performance
Source: PLoS Biol. 2022 Mar 17;20(3):e3001560. doi: 10.1371/journal.pbio.3001560 (PMC8929635; doi:10.1371/journal.pbio.3001560)

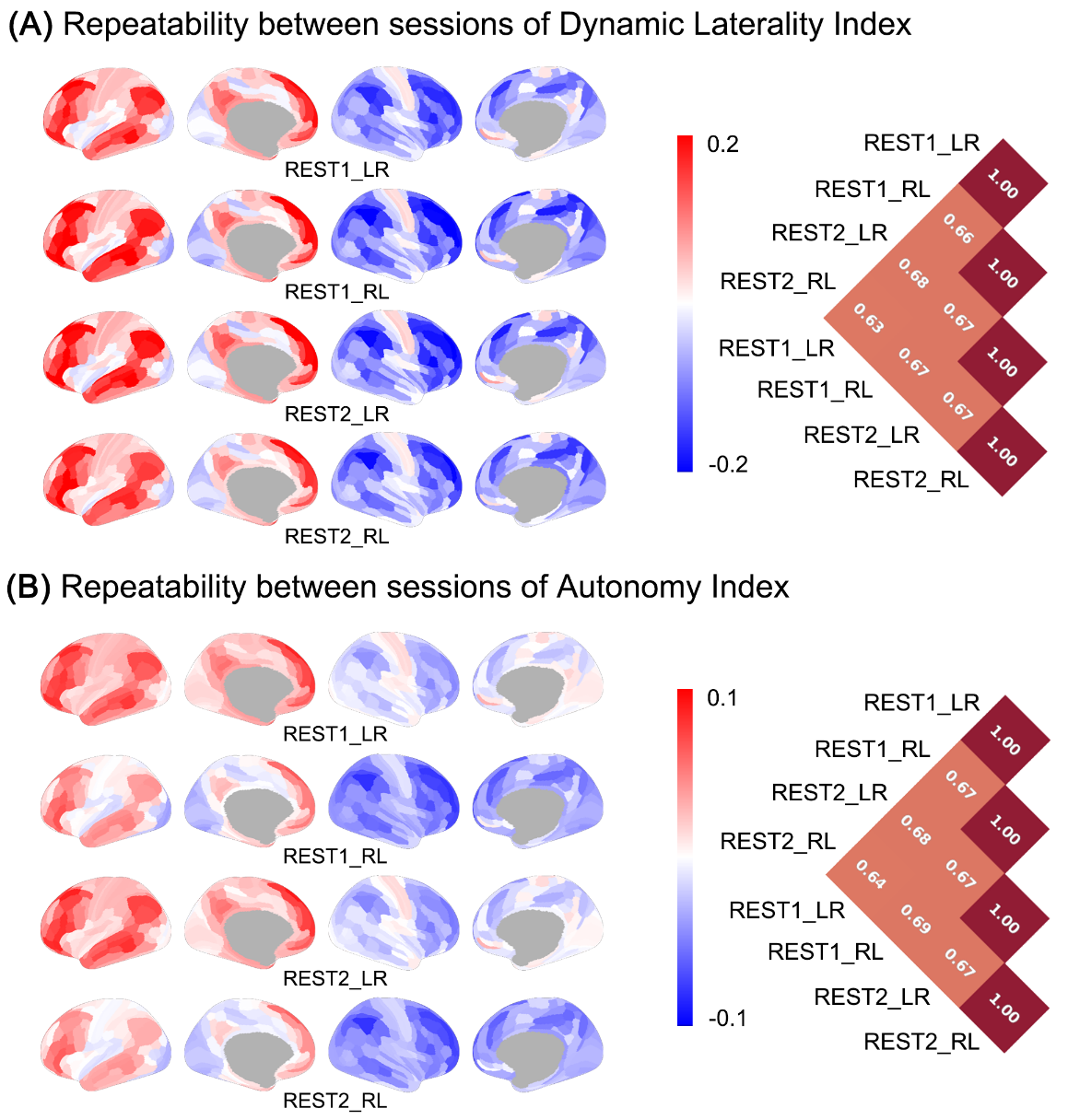

Supplement: S1 Fig — Notice that the spatial distribution of MLI and AI is very similar. (A) Left: the average DLI map of 4 sessions of HCP data. Right: correlation coefficient among the 4 sessions (average among all the participants). (B) Left: the AI map calculated by 4 sessions of HCP data. Right: correlation coefficient between the 4 sessions (average among all the participants). The underlying data for this figure can be found in S5 Data. AI, autonomy index; DLI, dynamic laterality index; HCP, Human Connectome Project; MLI, mean laterality index. (PNG) [file pbio.3001560.s001.png]

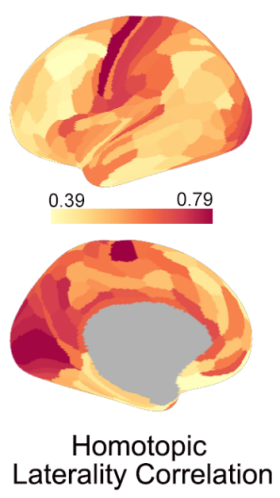

Supplement: S2 Fig — (PNG) [file pbio.3001560.s002.png]

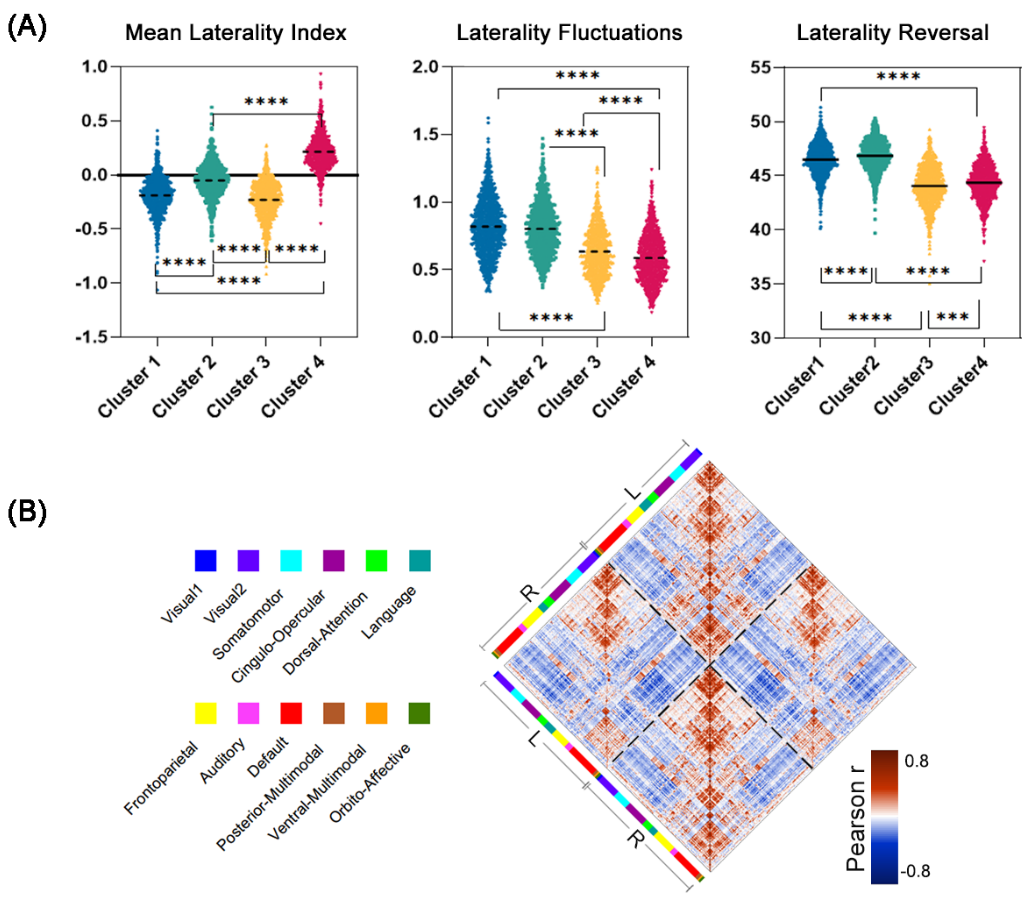

Supplement: S3 Fig — (A) Dynamic laterality measures (MLI, LF, and LR) across 4 clusters. The statistical test was repeated-measure ANOVA, with the asterisk representing significance (***, p < 0.01, ****, p <0.001) (B) The averaged laterality correlation matrix over all 991 participants (arranged by 12 functional networks of the CAB-NP). The underlying data for this figure can be found in S5 Data. CAB-NP, Cole-Anticevic Brain-wide Network Partition; LF, laterality fluctuations; LR, laterality reversal; MLI, mean laterality index. (PNG) [file pbio.3001560.s003.png]

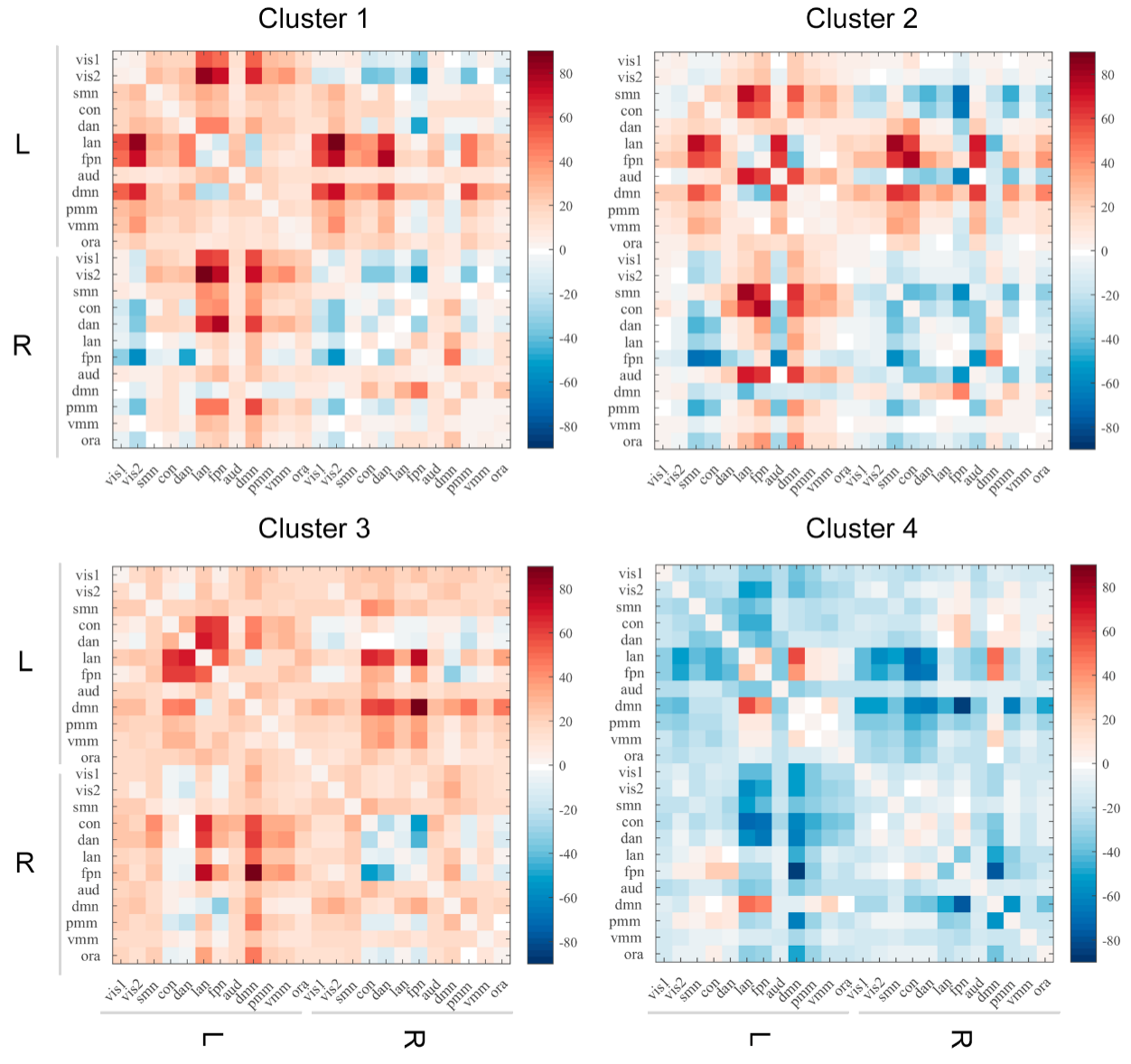

Supplement: S4 Fig — We adopted the CAB-NP that contains 12 pairs of networks in bilateral hemispheres. We calculated the dynamic FC within/between the 12 pairs of networks, and then calculated their regression coefficients of DLI on FC for each participant in each run. We averaged the regression coefficients of the 4 runs of each participant and tested whether they were significantly greater than or less than 0 with single-sample t test. The figure shows the t-values of each functional connectivity. Vis1, Visual1; vis2, Visual2; smn, Somatomotor; con, Cingulo-Opercular network; dan, Dorsal-Attention network; lan, Language network; fpn, Frontoparietal network; aud, Auditory network; dmn, Default Mode network; pmm, Posterior-Multimodal; vmm, Ventral-Multimodal; ora, Orbito-Affective. L, left hemisphere; R, right hemisphere. Note: This figure is the same as Fig 5 in the text, but it shows the regression coefficients of all FC (without thresholds) with more detail. The underlying data for this figure can be found in S4 Data. CAB-NP, Cole-Anticevic Brain-wide Network Partition; DLI, dynamic laterality index; FC, functional connectivity. (PNG) [file pbio.3001560.s004.png]

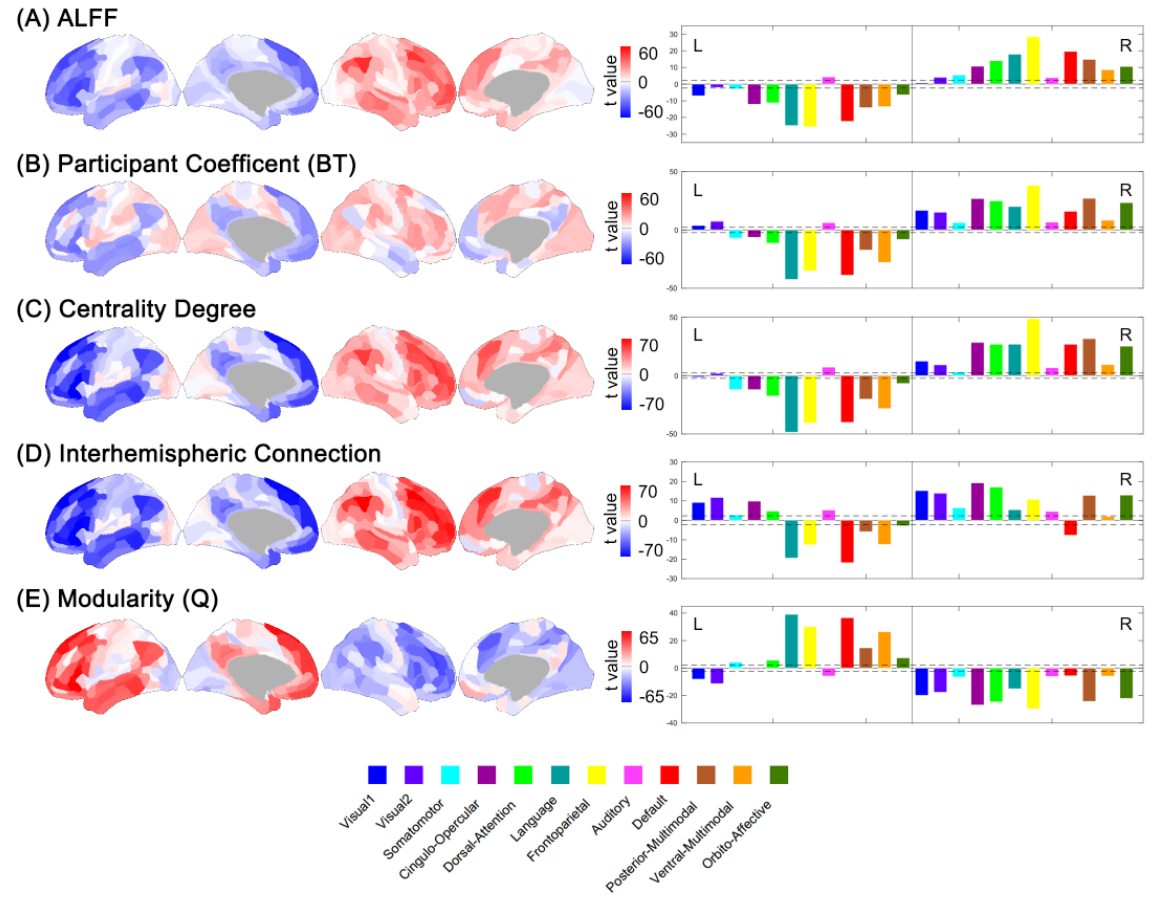

Supplement: S5 Fig — (A-E) The association between the DLI and the average time series of network indicators and ALFFs of each brain region and each subnetwork. The values in the brain maps represent the t-value of the Pearson correlation coefficient r (one-sample t test). The underlying data for this figure can be found in S5 Data. ALFF, amplitude of low-frequency fluctuation; DLI, dynamic laterality index. (PNG) [file pbio.3001560.s005.png]

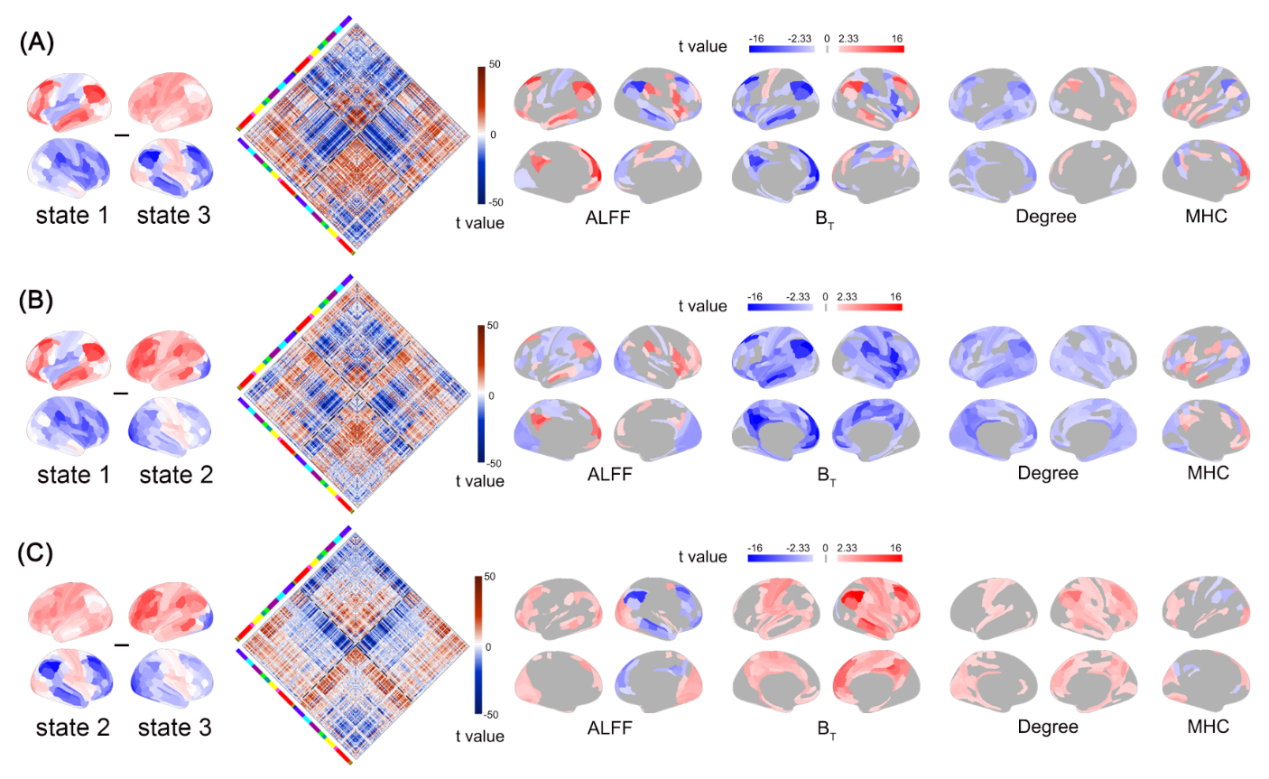

Supplement: S6 Fig — The paired t test was used, and all values shown in the figure were t statistics. Only regions with t-value exceeded the significant threshold (±2.33, p < 0.001) were shown in brain maps. The underlying data for this figure can be found in S5 Data. ALFF, amplitude of low-frequency fluctuation. (PNG) [file pbio.3001560.s006.png]

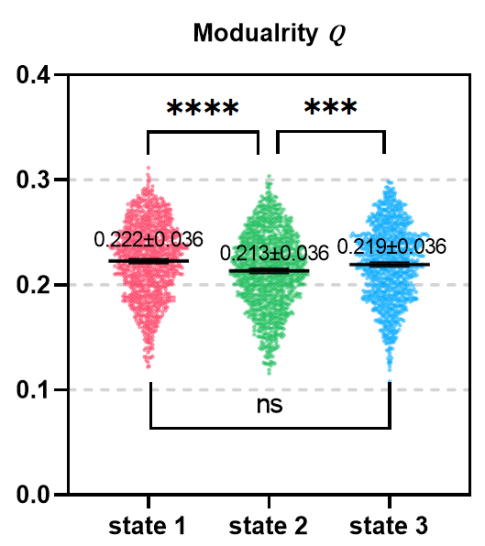

Supplement: S7 Fig — The one-way ANOVA and post hoc test were used. One asterisk (*), p < 0.05; 2 asterisks (**), p < 0.01; 3 asterisks (***), p < 0.001; 4 asterisks (****), p < 0.0001; ns, nonsignificant. The underlying data for this figure can be found in S5 Data. (PNG) [file pbio.3001560.s007.png]

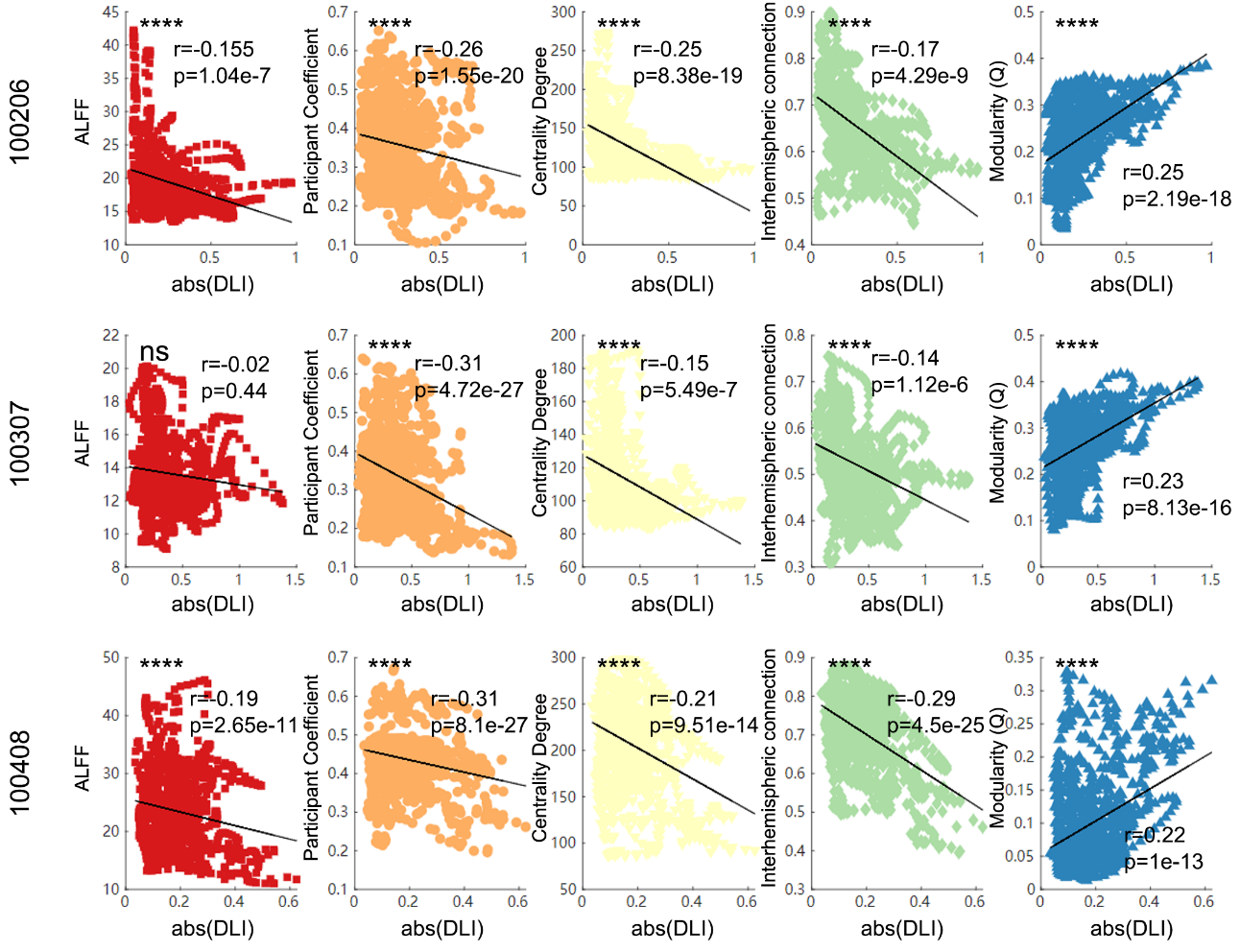

Supplement: S8 Fig — Higher absolute value represents higher left/right lateralization. We selected data from 3 participants, sub-100206, sub-100307, and sub-100408, to show the relationship between global lateralization (quantified by the averaged absolute value of laterality) and dynamic ALFF/graph theory indicators. The results show that higher global lateralization is associated with lower ALFF and stronger whole-brain dissociation (higher modularization Q, lower participant coefficient, centrality degree, and interhemispheric connectivity). One asterisk (*), p < 0.05; 2 asterisks (**), p < 0.01; 3 asterisks (***), p < 0.001; 4 asterisks (****), p < 0.0001; ns, nonsignificant. The underlying data for this figure can be found in S5 Data. ALFF, amplitude of low-frequency fluctuation; DLI, dynamic laterality index. (PNG) [file pbio.3001560.s008.png]

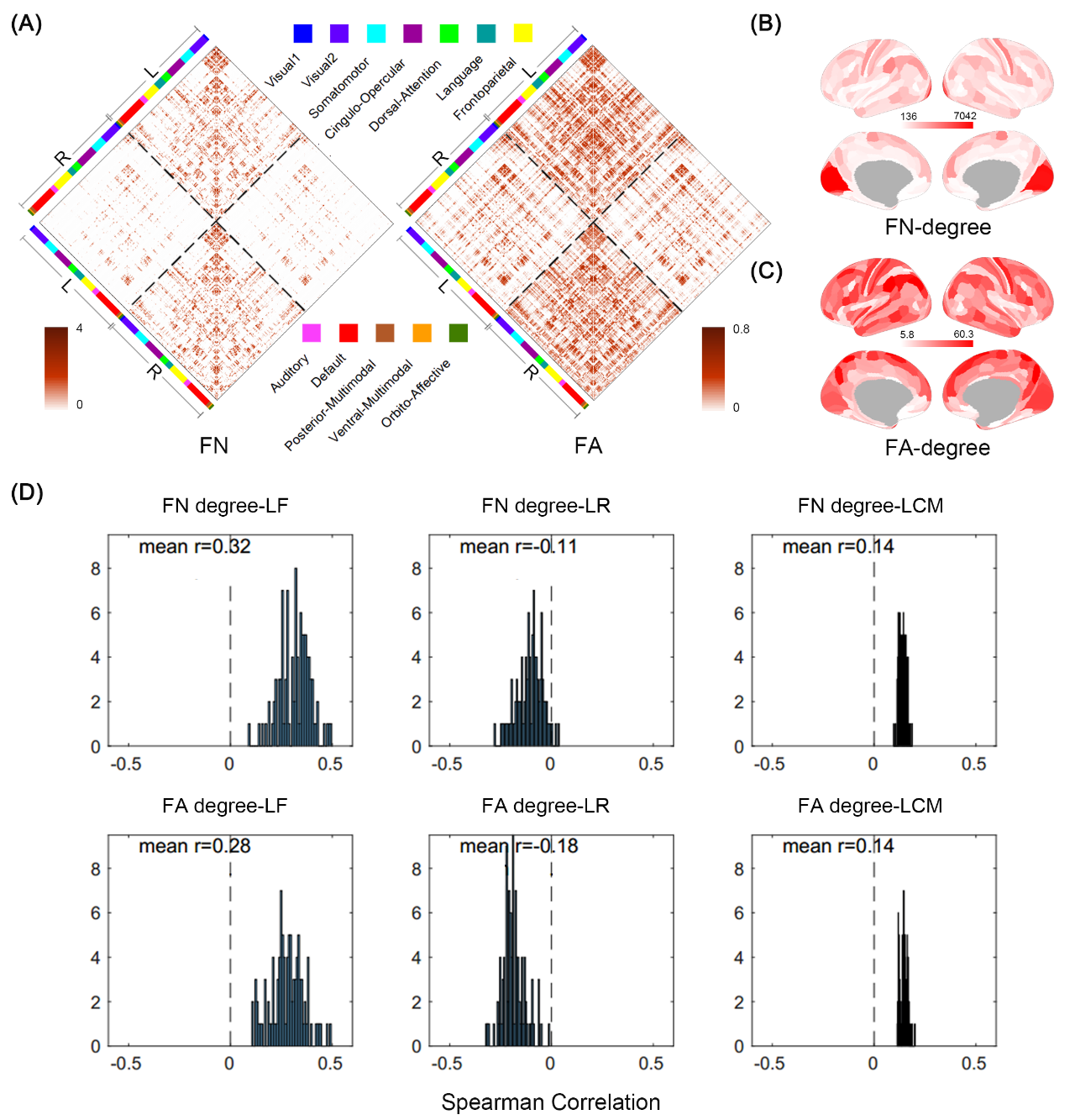

Supplement: S9 Fig — (A) Mean structural connection matrix. (B) Mean FN degree. (C) FA degree. (D) The distribution of Spearman correlation coefficients between structural connection attributes [FN-degree, FA-degree, and SCM (based on FN or FA)] and dynamic laterality measures (LF, LR, and LCM). FA, fractional anisotropy; FN, fiber number; LCM, laterality correlation matrix; LF, laterality fluctuations; LR, laterality reversal; SCM, structural connection matrix. (PNG) [file pbio.3001560.s009.png]

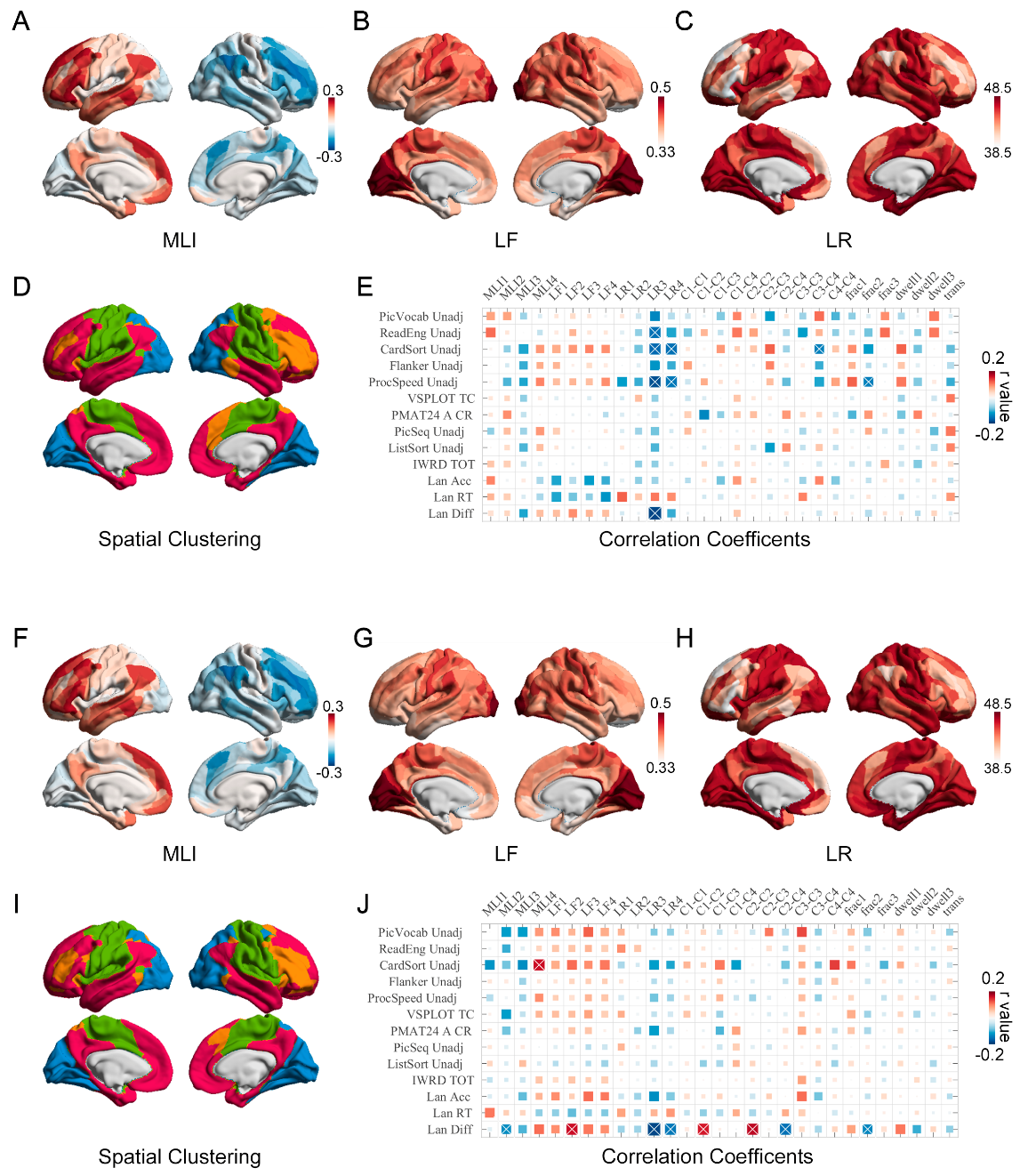

Supplement: S10 Fig — (A-E) Results of part 1 (odd indexed participants in participant list, N = 446). (F-G) Results of part 2 (even indexed participants in participant list, N = 445). (A/F) MLI. (B/G) LF. (C/H) LR. (D/I) Result of spatial clustering. (E/J) Correlation between cognitive measures and indicator of dynamic lateralization. The cross indicates that P is less than 0.05 (FDR). C1-C4: correlation between DLI of Cluster 1 and Cluster 4, and so on. DLI, dynamic laterality index; FDR, false discovery rate; LF, laterality fluctuations; LR, laterality reversal; MLI, mean laterality index. (PNG) [file pbio.3001560.s010.png]

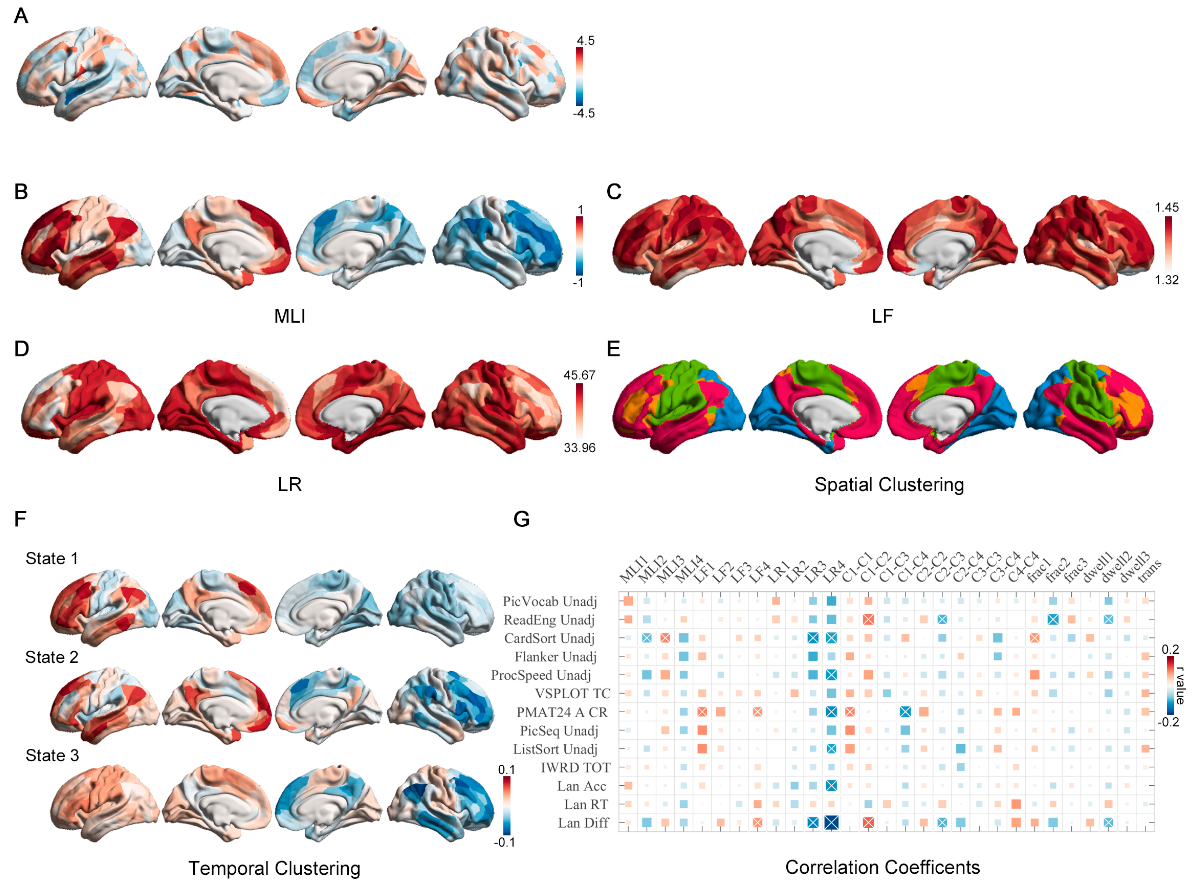

Supplement: S11 Fig — (A) The t-value of regression coefficient of DLI on GS. (B) MLI. (C) LF. (D) LR. (E) Result of spatial clustering. (F) The results of temporal clustering. (G) Correlation between cognitive measures and indicator of dynamic lateralization. The cross indicates FDR q < 0.05. C1-C4: correlation between DLI of Cluster 1 and Cluster 4, and so on. DLI, dynamic laterality index; FDR, false discovery rate; GS, global signal; LF, laterality fluctuations; LR, laterality reversal; MLI, mean laterality index. (PNG) [file pbio.3001560.s011.png]

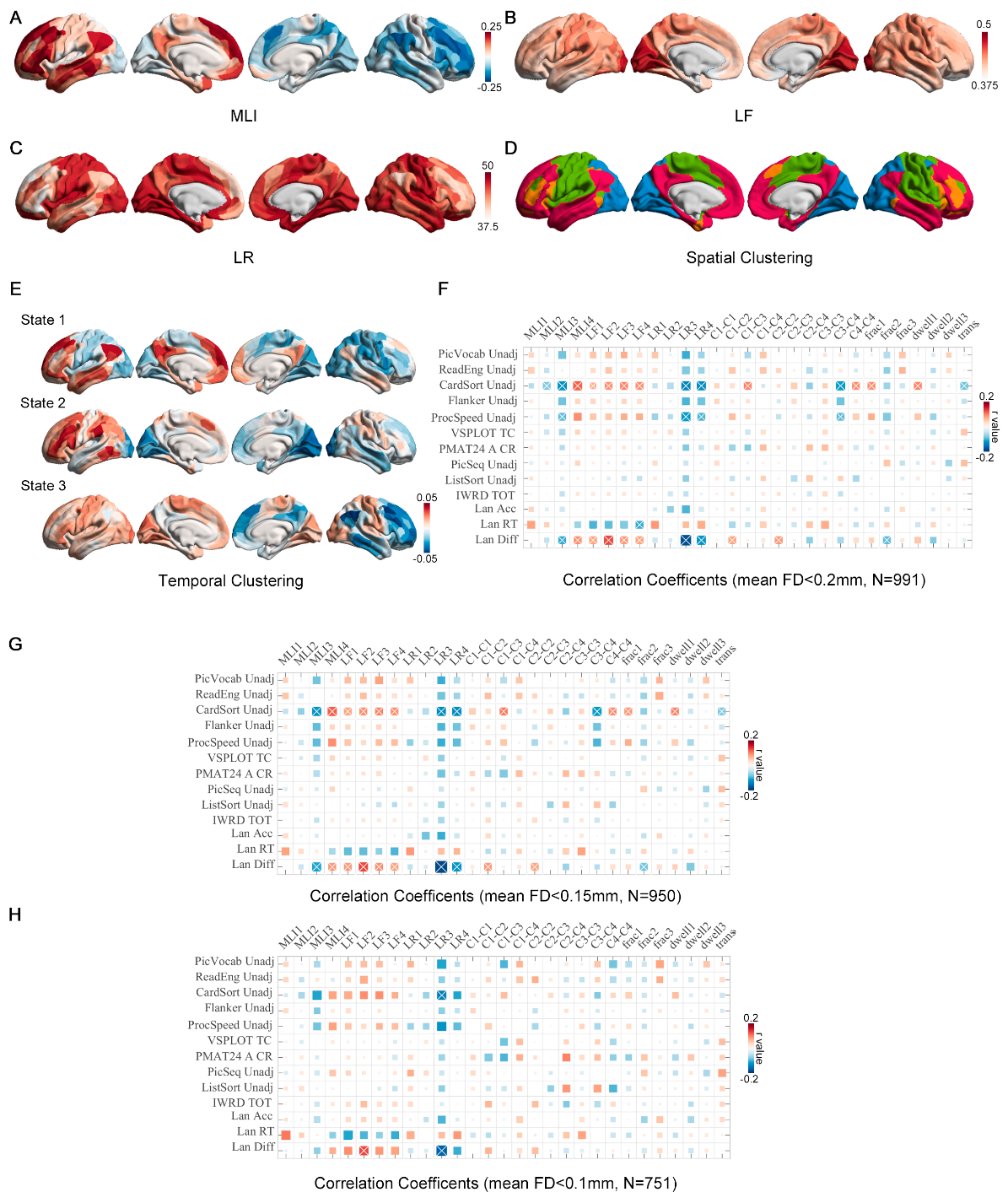

Supplement: S12 Fig — (A-F) Results using BOLD residual (with FD and Friston 24 parameters being regressed out). (A) MLI. (B) LF. (C) LR. (D) Result of spatial clustering. (E) Results of temporal clustering. (G-H) Correlation between cognitive measures and DLI. The cross indicates FDR q < 0.05. C1-C4: correlation between DLI of Cluster 1 and Cluster 4, and so on. (G) Correlation results using participants of mean FD < 0.15 mm. (H) Correlation results using participants of mean FD < 0.1 mm. DLI, dynamic laterality index; FD, frame distance; FDR, false discovery rate; LF, laterality fluctuations; LR, laterality reversal; MLI, mean laterality index. (PNG) [file pbio.3001560.s012.png]

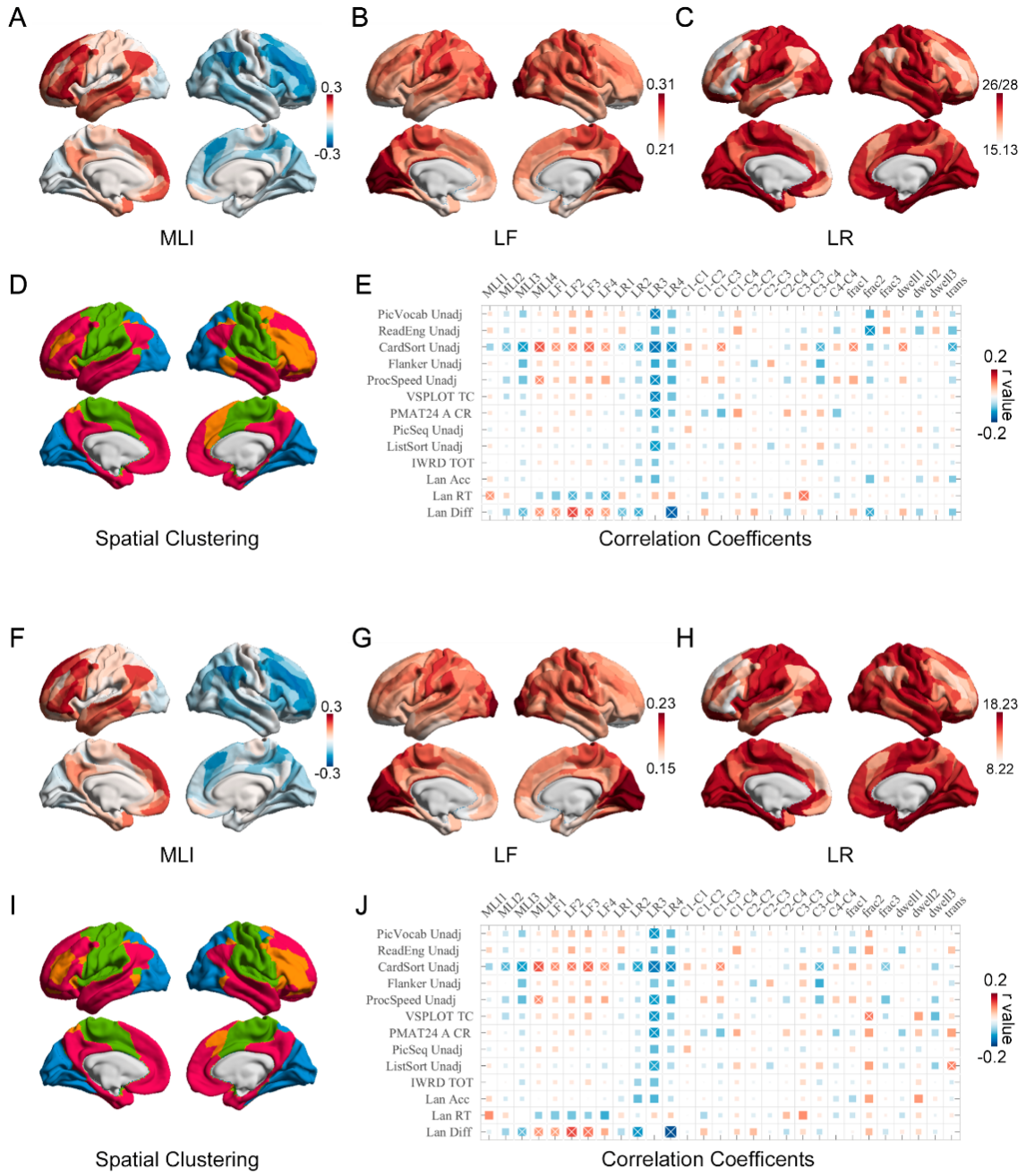

Supplement: S13 Fig — (A-E) Results of window size = 60 TRs. (F-G) Results of window size = 90 TRs. (A/F) MLI. (B/G) LF. (C/H) LR. (D/I) Result of spatial clustering. (E/J) Correlation between cognitive measures and DLI. The cross indicates that P is less than 0.05 (FDR corrected). C1-C4: correlation between DLI of Cluster 1 and Cluster 4, and so on. DLI, dynamic laterality index; FDR, false discovery rate; LF, laterality fluctuations; LR, laterality reversal; MLI, mean laterality index. (PNG) [file pbio.3001560.s013.png]

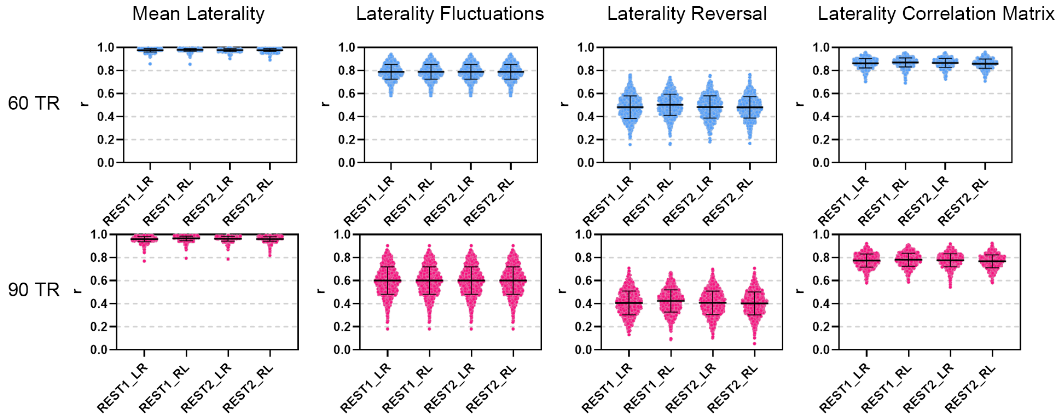

Supplement: S14 Fig — The r on vertical axis represents Pearson correlation coefficient between dynamic laterality indictor patterns using window length of 60 TR/90 TR and indictor patterns of 30 TR (used in the text) among all participants. Each dot in the graph represents one of the 991 participants. As can be seen, MLI has the highest reproducibility (close to 1), followed by laterality correlation, LF, and LR. With the increase of the window length, the correlation between the dynamic laterality indicators and the results of the window length of 30 TR decreased gradually. REST1/REST2 indicates the scan time (in first or second day). LR/RL indicates the scanning direction. LR, left to right. RL, right to left. LF, laterality fluctuations; LR, laterality reversal; MLI, mean laterality index. (PNG) [file pbio.3001560.s014.png]

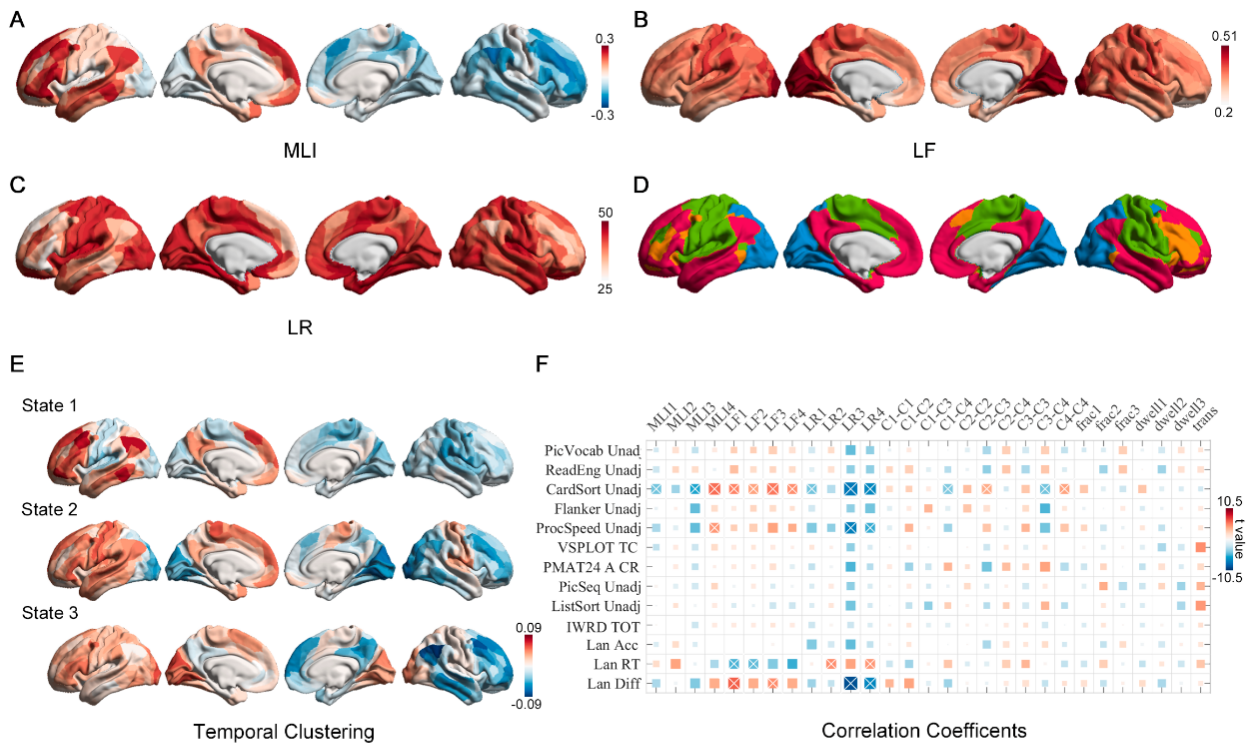

Supplement: S15 Fig — (A-F) The influence of removing the ROI from its ipsilateral GS in calculating the laterality index. That is, we reconstructed GS by removing the ROI A from its ipsilateral GS. (A) MLI. (B) LF. (C) LR. (D) Result of spatial clustering. (E) The results of temporal clustering. (F) Correlation between cognitive measures and indicator of dynamic lateralization. The cross indicates FDR q < 0.05. C1-C4: correlation between DLI of Cluster 1 and Cluster 4, and so on. DLI, dynamic laterality index; FDR, false discovery rate; GS, global signal; LF, laterality fluctuations; LR, laterality reversal; MLI, mean laterality index; ROI, region of interest. (PNG) [file pbio.3001560.s015.png]
