# Supplementary material for: Dynamic changes in brain lateralization correlate with human cognitive performance
Source: PLoS Biol. 2022 Mar 17;20(3):e3001560. doi: 10.1371/journal.pbio.3001560 (PMC8929635; doi:10.1371/journal.pbio.3001560)
Supplement: S1 Text — (DOCX) [file pbio.3001560.s024.docx]

**Supplemental Text**

*The relationship between* *Global-signal based Dynamic Laterality Index (DLI) and Autonomy Index (AI).*

The traditional laterality index, Autonomy Index (Chen et al., 2019; Liu, Stufflebeam, Sepulcre, Hedden, & Buckner, 2009; Mueller, Wang, Pan, Holt, & Liu, 2015; Wang, Buckner, & Liu, 2014) calculates the mean correlation coefficient (functional connectivity) between a ${ROI}_{y}$ and all regions ($x_{i}$) of the left and the right hemisphere ($\bar{r}_{xy}=\frac{\sum_{i=1}^{n} r_{x_{i}y}}{n}$), respectively, and used the difference between these two mean correlation coefficients as a measure of an ROI’s laterality. In comparison, our laterality index directly calculates the correlation coefficient between ${ROI}_{y}$ and the global signal from the left and the right hemisphere ($r_{\bar{x}y}$, where $\bar{x}=\frac{\sum_{i=1}^{n} x_{i}}{n}$), and then calculated the difference between the two correlation coefficients as the ROI’s laterality.

We analyzed in the following that these two indices are intrinsically related, by showing that the mean correlation coefficient between ${ROI}_{y}$ and regions in one hemisphere is correlated to the correlation coefficient between ${ROI}_{y}$ and the global signal of that hemisphere.

$$\bar{r}_{xy}=\frac{1}{n}\sum_{i=1}^{n} \frac{E\left[ \left( x_{i}-u_{x_{i}} \right)\left( y-u_{y} \right) \right]}{\delta x_{i}\delta y}=\frac{1}{n}\sum_{i=1}^{n} \frac{E\left[ x_{i}y \right]-E\left[ x_{i} \right]E\left[ y \right]}{\delta x_{i}\delta y}$$

$$r_{\bar{x}y}=\frac{E[\left( \bar{x}-u_{\bar{x}} \right)\left( y-u_{y} \right)]}{\delta\bar{x}\delta y}=\frac{E\left[ \bar{x}y \right]-E\left[ \bar{x} \right]E\left[ y \right]}{\sqrt{\delta^{2}\left( \frac{x_{1}+\ldots x_{n}}{n} \right)}\delta y}=\frac{E\left[ \bar{x}y \right]-E\left[ \bar{x} \right]E\left[ y \right]}{\frac{1}{n}\sqrt{\sum_{i=1}^{n} \delta^{2}x_{i}+\sum_{i=1}^{n} \sum_{i\neq j}^{n} Cov\left( x_{i},x_{j} \right)}\delta y}$$

Where $E$ stands for expectation and $\delta$ for standard deviation. It should be noted that only when all $x_{i}$ are independent with each other can $\bar{r}_{xy}$ and $r_{\bar{x}y}$ be highly correlated. For real fMRI data, $x_{i}$s are dependent. Therefore, we also calculated the correlation between AI and DLI based on empirical data to jointly prove the consistency between our indicators and previous indicators (see Methods).

*Results of Validation Analyses*.

*(a) The spilt-half validation.*

All 991 subjects were spilt into two halves (N_part1_=446, N_part2_=445) and the main analyses were repeated separately on these two halves of the data. Specifically, we lined all the subjects together according to their subject ID and divided their odd and even rows as two samples (part 1 and part 2). We found that the averaged maps of the two samples for MLI/LF/LR are highly similar, and the community structure obtained was completely consistent with the results obtained using all samples, indicating that the samples had little influence on the basic characteristics of DLI (S10 Fig).

We then calculated the correlation between dynamic laterality and behavioral measures in the two samples, respectively. At uncorrected p<0.05 level, higher cognitive flexibility (Card Sort Task) and better language ability (Story Difficulty of Language Task) were all associated with higher LF and lower LR of the Cluster 3 and Cluster 4 (for CardSort, part 1: Cluster 3 LF, r=0.13, p=0.004; LR, r=-0.179, p=0.0001; Cluster 4 LF, r=0.12, p=0.009; LR, r=-0.172, p=0.0002; part 2: Cluster 3 LF, r=0.114, p=0.01; LR, r=-0.12, p=0.009; Cluster 4 LF, r=0.12, p=0.009; LR, r=-0.118, p=0.01; for LanDiff, part 1: Cluster 3 LF, r=0.1, p=0.03; LR, r=-0.205, p=9.94e-6; Cluster 4 LF, r=0.1, p=0.034; LR, r=-0.13, p=0.0072; part 2: Cluster 3 LF, r=0.13, p=0.0046; LR, r=-0.196, p=2.78e-5; Cluster 4 LF, r=0.12, p=0.01; LR, r=-0.16, p=0.0006). Most of the correlations were weaker than those obtained for all 991 subjects, possibly due to the smaller sample size.

*(b) Controlling the effect of head movements.*

We quantified the effect of head movements using linear regression models of DLI on head movement across time windows and repeated the main analyses using DLI residuals (with head movement being regressed out, see S12 Fig A-F). Specifically, for each brain area of each subject in each run, we constructed a linear regression model using 24 head movement parameters (Friston’s 24 motor parameter (Friston, Williams, Howard, Frackowiak, & Turner, 1996) in each time window) as the independent variable, and DLI time series of this brain area as the dependent variable. The distribution of dynamic laterality indices (MLI/LF/LR/spatial clusters/temporal clusters) was similar with our main results showed in text (S12 Fig A-E), and their correlation with cognitive performance were largely replicated: Higher CardSort/ProcSpeed/LanDiff showed assocation with higher LF and lower LR of the Cluster 3 and Cluster 4 (S12 Fig F).

We also used more stringent exclusion criteria (mean FD<0.15mm and FD<0.1mm) to repeat our analyses. The association between cognitive performance and dynamic laterality indices has weakened, but the pattern remains the same (higher CardSort/ProcSpeed/LanDiff were associated with higher LF and lower LR of the Cluster 3 and Cluster 4, see S12 Fig G-H).

*(c) Controlling the effect of global signal.*

We repeated the main analyses with the global signal of the whole brain being regressed out. Specifically, for each brain area of each run of each subject, we constructed a linear regression model, using global signal as the independent variable, and DLI time series of this brain area as the dependent variable to obtain a regression coefficient beta. All results were corrected by FDR (q<0.05). We found that the effect of global signal to the DLI is not significant (S11 Fig A).

Besides, we also repeated our analysis using data with global signal of the whole brain being regressed out and found GS does not influence our results significantly: the distribution of MLI, LF, LR, spatial clusters and temporal clusters remain the same (S11 Fig B-F); CardSort, ProcSpeed and LanDiff showed positive correlation with LF while negative correlation with LF, specifically in Cluster 3 and 4 (S11 Fig G).

*(d) Controlling the effect of time window lengths.*

Different time window lengths (60 TRs and 90 TRs) were used to investigate the reproducibility of our results. We found that a window length from 60 TR to 90 TR yield quite similar results and the brain-behavior correlation: the distribution of MLI, LF, and LR cross the brain, the spatial clusters and temporal clusters did not change significantly (S13 Fig B-F); CardSort, ProcSpeed and LanDiff showed positive correlation with LF while negative correlation with LR, specifically in Cluster 3 and 4 (S13 Fig). All results were corrected by FDR (q<0.05).

In addition, we also calculated the Pearson correlation coefficient between indictor pattern using window length of 60TR/90TR and indictor pattern of 30TR (used in the text) among each subject. MLI showed the highest reproducibility (close to 1), followed by laterality correlation, LF, and LR. Repeatability varies very little between sessions. With the increase of the window length, the correlation between the dynamic laterality indicators and the results of the window length of 30TR decreased gradually. LR, which had the lowest repeatability, showed an average repeatability of 0.4 when the window length is 90TR. see S14 Fig for more details.

*(e) Controlling the effect of the contaminating effect of ROI to its ipsilateral GS.*

The potential contaminating effect of ROI to its ipsilateral GS was analyzed. When we calculated the correlation coefficient between an ROI *A* and the global signal of its ipsilateral hemisphere, the ROI *A* was included in its ipsilateral hemisphere global signal. Thus, the global signal may be "contaminated" by the activity of the ROI *A*. Therefore, we removed the ROI *A* when extracting its ipsilateral hemisphere global signal and repeated our main analyses to examine whether our results would be affected. Specifically, we re-constructed GS' by removing the ROI *A* from its ipsilateral hemisphere global signal and then calculated the correlation between ROI *A* and ipsilateral GS'. We found no significant change in the results, indicating that the contaminating effect may be limited (S15 Fig).

**Reference**

Chen, Q., Beaty, R. E., Cui, Z., Sun, J., He, H., Zhuang, K., . . . Qiu, J. (2019). Brain hemispheric involvement in visuospatial and verbal divergent thinking. *Neuroimage, 202*, 116065.

Friston, K. J., Williams, S., Howard, R., Frackowiak, R. S. J., & Turner, R. (1996). Movement‐related effects in fMRI time‐series. *Magnetic resonance in medicine, 35*(3), 346-355.

Liu, H., Stufflebeam, S. M., Sepulcre, J., Hedden, T., & Buckner, R. L. (2009). Evidence from intrinsic activity that asymmetry of the human brain is controlled by multiple factors. *Proceedings of the National Academy of Sciences of the United States of America, 106*(48), 20499-20503.

Mueller, S., Wang, D., Pan, R., Holt, D. J., & Liu, H. (2015). Abnormalities in Hemispheric Specialization of Caudate Nucleus Connectivity in Schizophrenia. *JAMA Psychiatry, 72*(6), 552. doi:10.1001/jamapsychiatry.2014.3176

Wang, D., Buckner, R. L., & Liu, H. (2014). Functional Specialization in the Human Brain Estimated By Intrinsic Hemispheric Interaction. *Journal of Neuroscience, 34*(37), 12341-12352.
