# Supplementary material for: Dynamic changes in brain lateralization correlate with human cognitive performance
Source: PLoS Biol. 2022 Mar 17;20(3):e3001560. doi: 10.1371/journal.pbio.3001560 (PMC8929635; doi:10.1371/journal.pbio.3001560)
Supplement: Attachment — Submitted filename: Response to Reviewers PlosBio 20211104.docx [file pbio.3001560.s026.docx]

Dear Editor Smith,

First, we thank you very much for your timely processing of our manuscript. We are also encouraged by the positive comments of all 3 reviewers and quite grateful for their valuable suggestions which allowed us to improve our work significantly. All the revisions made are marked in red in the revised manuscript. Below is a point-by-point response to all reviewers’ comments.

**Reviewer #1**: The authors report analyses of resting state fMRI data from the Human Connectome Project examining brain laterality dynamics and relationships with neurocognitive factors. This is a timely topic and an interesting and revealing set of analyses. Some specific comments are below.

Reply

We thank the reviewer very much for the positive comments of our work.

In the introduction, the authors may want to see some earlier empirical FC studies that have examined intrinsic interhemispheric relationships:

Low frequency fluctuations reveal integrated and segregated processing among the cerebral hemispheres. Gee DG, Biswal BB, Kelly C, Stark DE, Margulies DS, Shehzad Z, Uddin LQ, Klein DF, Banich MT, Castellanos FX, Milham MP. Neuroimage. 2011 Jan 1;54(1):517-27.

Regional variation in interhemispheric coordination of intrinsic hemodynamic fluctuations. Stark DE, Margulies DS, Shehzad ZE, Reiss P, Kelly AM, Uddin LQ, Gee DG, Roy AK, Banich MT, Castellanos FX, Milham MP. J Neurosci. 2008 Dec 17;28(51):13754-64.

Growing together and growing apart: regional and sex differences in the lifespan developmental trajectories of functional homotopy. Zuo XN, Kelly C, Di Martino A, Mennes M, Margulies DS, Bangaru S, Grzadzinski R, Evans AC, Zang YF, Castellanos FX, Milham MP. J Neurosci. 2010 Nov 10;30(45):15034-43.

Reply

We thank the reviewer very much for the references recommended, i.e., earlier FC studies that were related to intrinsic interhemispheric relationship, which we have cited in the revised manuscript, see page 3.

The authors introduce two measures of laterality dynamics - laterality fluctuations and laterality reversal. Can the authors provide some background and rationale for why these specific measures were employed? How are they thought to index and/or relate to the cognitive laterality aspects that are reviewed in the introduction?

Reply

In fact, the standard deviation of a time series is commonly used in dynamic brain network analysis, which reflects the variability of the measure ^1-4^, e.g., the standard deviation of time-varying functional connectivity reflects its variability over time. In a similar manner we also calculated the standard deviation of dynamic laterality time series, to reflect the level of fluctuations of laterality over time.

Laterality reversal is proposed based on the empirical findings that a region may change its directions of laterality over time (i.e., left- or right lateralized). This is also inspired by dynamic brain network analysis, i.e., a region may change the module it belongs to (named "nodal flexibility" proposed by Bassett et al.), which correlated closely with cognitive ability ^5,6^. Similarly, if we regard the two hemispheres as two large functional modules, laterality reversal thus could be considered as the ability of a region to switch between these two larger modules.

In the MS, we have also considered the relationship between Laterality fluctuation and Laterality reversal, i.e., “Laterality reversal suggests that laterality of a region fluctuate too much and deviates remarkably from the mean value (the dominant laterality regime, Fig. 1d), i.e., a region’s functional connectivity switches from being more ipsilateral to highly contralateral” (see page 12). We have added some explanations about the background and a rationale of the method in the **Results** section in the new manuscript (page 5).

How might the DLI be influenced by the fact that the global signal does not display a uniform topography across the brain? See for example:

Topography and behavioral relevance of the global signal in the human brain.

Li J, Bolt T, Bzdok D, Nomi JS, Yeo BTT, Spreng RN, Uddin LQ. Sci Rep. 2019 Oct 3;9(1):14286.

Reply

We thank the reviewer for this advice. We performed a correlation analysis between global signal and DLI and found that the effect of global signal to the DLI is not significant (Supplemental Figure 10A). We have added some discussion of this problem with the reference being cited (page 14).

Besides, we also repeated our analysis with global signal being regressed out and found that GS does not influence our results significantly: the distributions of MLI, LF, LR, spatial clusters and temporal clusters remain largely unchanged (Supplemental Figure 10B-F); CardSort, ProcSpeed and LanDiff still showed positive correlation with LF while negative correlation with LF, specifically in Cluster 3 and 4 (Supplemental Figure 10G). We have added this figure in the Supplemental Materials (Supplemental Figure 10).

*Supplemental Figure 10. The result with global signal regressed out. (A) the effect of global signal to DLI (t value of regression coefficient). (B) mean laterality index (MLI). (C) laterality fluctuation (LF). (D) laterality reversal (LR). (E) result of spatial clustering. (F) the results of timing clustering. (G) correlation between cognitive measures and indicator of dynamic lateralization. The cross indicates that P is less than 0.05 (uncorrected).* *C1-C4: correlation between dynamic laterality index of Cluster 1 and Cluster 4, and so on.*

How was the length of the sliding window determined (for the DLI computation)?

Reply

We adopted a window length of 0.72s×30TR=21.6s, referring to an article of Gonzalez-Castillo et al.^7^, who found window sizes as short as 22.5s was optimal to accurately track mental states. This adopted window length is slightly shorter than the time window commonly used (30-60s), as the time resolution of HCP data (0.72s) is much higher than that of common fMRI data (generally around 2s).

Moreover, we also tested the stability of the results with regard to different time window lengths (60TRs/43.2s, 90TRs/64.8s), with the results presented in the MS (page 10) and Supplemental Results: “We found that a window length from 60 TR to 90 TR would yield quite similar results and the brain-behavioral correlation”. Supplemental Fig.13 A-E showed results with window size=60TRs and Supplemental Fig. 13F-J showed results with window size=90TRs. A/B/C/D and F/G/H/I showed highly similar patterns. CardSort, ProcSpeed and LanDiff showed positive correlation with LF while negative correlation with LF, specifically in Cluster 3 and 4 (Supplemental Fig. 13E&J).

*Supplemental Figure 13. The result of different window size. (A-E) results of window size=60TRs. (F-J) results of window size=90TRs. (A/F) mean laterality index (MLI). (B/G) laterality fluctuation (LF). (C/H) laterality reversal (LR). (D/I) result of spatial clustering. (E/J) correlation between cognitive measures and indicator of dynamic lateralization. The cross indicates that P is less than 0.05 (FDR). C1-C4: correlation between dynamic laterality index of Cluster 1 and Cluster 4, and so on.*

It is not clear what the analyses utilizing ALFF are meant to convey.

Reply

Larger ALFF is thought to be related to the larger magnitude of neural activity, which generally means higher energy consumption in low-frequency regional brain activity^8,9^. In view of the previous literatures that have pointed out that segregation processing is a more economic and low-energy-consumptive (since information can be processed within local module rather than to be transmitted across different modules)^10-12^, we therefore investigated whether higher level of lateralization across the brain in a time window is also associated with lower magnitude of energy consumption (i.e. ALFF). We found such a negative correlation between ALFF and averaged absolute value of DLI, suggesting higher lateralization is related to reduced energy consumption.

Finally, we would like to thank the 1^st^ reviewer again for the positive comments to our work and the suggestions that allow us to improve our MS and clarity.

**Reviewer #2**: The authors asked the interesting question of whether hemispheric lateralization, which is thought to be a stable trait-like feature, fluctuates over short time scales. They use resting-state data from the Human Connectome Project and a novel method of measuring dynamic functional connectivity that they call the dynamic lateralization index (DLI). The others argue that hemispheric lateralization is adaptive and dynamic changes to lateralization in brain networks are correlated to cognitive tasks, brain network properties, and heritability. While these results are likely to be of interest to a wide audience of neuroscientists, I have major concerns about the methods that cast doubt over all of the results.

Reply

We thank the reviewer very much for the positive comments of our work such as “interesting question”, and “likely to be of interest to a wide audience of neuroscientists”.

1. The authors ignore prior work that quite convincingly shows that correlations in dynamic functional connectivity of BOLD fluctuations within small sliding time windows of resting-state data can be largely explained by sampling variability and head motion (Laumann et al., Cerebral Cortex, 2016). While I salute the authors attempt to validate the DLI, they do not go far enough in showing that the results are reliable. The first thing that they need to do is demonstrate that the initial calculation of the DLI (i.e., the correlations between the global signal of each hemisphere and ROI) is reliable. Probably the most efficient way to do so is to split the data in half and show that the DLI timeseries replicate across halves of participants. The large N (991 participants) is ideal for this type of replicability test. The authors should note that this is different from calculating the DLI and then averaging over the resultant timeseries (what they call the MLI) before comparing the results across runs. The important thing to demonstrate is that the patterns across the timeseries are stable.

Reply

We are quite thankful to the reviewer who pointed out our ignorance of the prior work, the validation of the dynamic laterality results and addressing the influence of head movements. Following these suggestions, we have cited the work mentioned by the reviewer, see page 14: “In view of the influence of global signal and head movements on the dynamic brain network statistics^13,14^”. We then conducted the spilt-half validation to test the repeatability of the dynamic laterality index.

Our analyses showed that our previous results are generally validated across two halves of the whole sample (based on whether the Subject Number was odd or even, the data was split into two parts: N_part1_=446, N_part2_=445). The spatial distribution of dynamic laterality measures and the community structure remained stable across samples (please see Supplemental Figure 9A-D and 9F-I). The p value of correlation between CardSort/LanDiff (cognitive performance) and LF/LR of Cluster 3 and 4 in both samples is less than 0.05.

For Card Sort, part 1: Cluster 3 LF, r=0.13, p=0.004; LR, r=-0.179, p=0.0001, FDR q<0.05; Cluster 4 LF, r=0.12, p=0.009; LR, r=-0.172, p=0.0002, FDR q<0.05. Part 2: Cluster 3 LF, r=0.114, p=0.015; LR, r=-0.12, p=0.0095; Cluster 4 LF, r=0.12, p=0.0066; LR, r=-0.118, p=0.013.

For LanDiff, part 1: Cluster 3 LF, r=0.1, p=0.03; LR, r=-0.205, p=9.94e-6, FDR q<0.05; Cluster 4 LF, r=0.1, p=0.034; LR, r=-0.13, p=0.0072. Part 2: Cluster 3 LF, r=0.13, p=0.0046; LR, r=-0.196, p=2.78e-5, FDR q<0.05; Cluster 4 LF, r=0.12, p=0.01; LR, r=-0.16, p=0.0006, FDR q<0.05, see Supplemental Fig. 9E&J.

We have added these results in the revised version (see page 10 in the MS and Supplemental Fig. 9 in the Supplemental Materials). Please see our response to the head movements problem in the next question.

*Supplemental Figure 9. The result of Spilt-half validation. (A-E) results of part 1 (subjects with odd number in subject list, N=446). (F-G) results of part 1 (subjects with even number, N=445). (A/F) mean laterality index (MLI). (B/G) laterality fluctuation (LF). (C/H) laterality reversal (LR). (D/I) result of spatial clustering. (E/J) correlation between cognitive measures and indicators of dynamic lateralization. The “cross” indicates that P is less than 0.05. C1-C4: correlation between dynamic laterality index of Cluster 1 and Cluster 4, and so on.*

Furthermore, the authors need to show more data related to head motion. While they mention that participants were excluded if they exceeded a motion threshold and they cleaned the remaining data using ICA-FIX, it is not clear whether the patterns of frame displacement in the remaining participants explains variance in the BOLD timeseries data. As is, I am not convinced that head motion is not still influencing the reported correlations.

Reply

We thank the reviewer for pointing out this important problem of head movements. In the previous submission we had removed the subjects with too large head motion (mean FD>0.2mm) to reduce the influence of head motion. In the revised manuscript, we furthermore removed the influence of multiple head motion parameters on BOLD following the suggestions of the reviewer. Specifically, FD and Friston 24 head motion parameters (the original value, first derivative and second derivative of displacement and rotation in the three directions of up-down, left-right, anterior-posterior, 24 parameters in total)^15^ were estimated using DPABI toolbox^16^ and then removed by linear regression models from BOLD time courses of each region.

We found that MLI/LF/LR, temporal and spatial clustering results remained largely unchanged (Supplemental Fig. 11A-E). The correlation results also basically keep uncharged (FDR corrected, q<0.05): LF of Cluster 3 and 4 still showed significant positive correlation with CartSort (Cluster 3: r=0.099, p=0.0027; Cluster 4: r=0.096, p =0.004), ProcSpeed (Cluster 3: r=0.074, p =0.026; Cluster 4: r=0.074, p =0.025) and LanDiff (Cluster 3: r=0.09, p =0.0066; Cluster 4: r=0.082, p =0.013). Meanwhile LR of Cluster 3 and 4 showed significant negative correlation with CartSort (Cluster 3: r=-0.13, p =4.9e-5; Cluster 4: r=-0.127, p=0.0001), ProcSpeed (Cluster 3: r=-0.11, p=0.0006; Cluster 4: r=-0.09, p=0.005) and LanDiff (Cluster 3: r=-0.175, p=1.14e-7; Cluster 4: r=-0.12, p=0.0003). See Supplemental Fig. 11F.

Besides, we also used more stringent exclusion criteria (mean FD<0.15mm / mean FD<0.1mm) to repeat the correlation analyses to investigate the influence of head motion on our results. We found that the results also remained generally unchanged under mean FD<0.15mm criterion, and the p-values of most of the results were still less than 0.05 under mean FD<0.1mm criterion, where the negative correlation between LR of Cluster 3, CardSort (r=-0.139, p=0.0003) and LanDiff (r=-0.16, p=1.7e-5) can pass FDR correction, see Supplemental Fig. 11G-H. We have added these results in the revised MS (page 10) and also the Supplemental Results.

*Supplemental Figure 11. (A-F) results with FD and Friston 24 parameters being regressed out. (A) mean laterality index (MLI). (B) laterality fluctuation (LF). (C) laterality reversal (LR). (D) result of spatial clustering. (E) the results of temporal clustering. (G-H) correlation between cognitive measures and indicator of dynamic lateralization. The cross indicates FDR q<0.05. C1-C4: correlation between dynamic laterality index of Cluster 1 and Cluster 4, and so on. (G) correlation results using subjects of mean FD<0.15mm. (H) correlation results using subjects of mean FD<0.1mm.*

In a nutshell, the authors should go back to the literature that casts doubt on observations of dynamic BOLD connectivity (like the paper mentioned above) and show that their data analysis method overcomes the major issues outlined in those papers. Otherwise, it is difficult to trust their results.

Reply

We totally agree with the reviewer, and we thank the reviewer very much for the suggestions that stimulate us to carry out a series of further analysis, to find out if head movements may have any effects on the dynamic laterality index. According to the suggestions of reviewers, we have included the results of addressing head movements effect in the Results (page 10) and Discussion section (page 14) and included results of split-half verification in the Results section (page 10).

2. The authors should also make the manuscript clearer in revision. A few things that stuck out me the most were:

1) in the first paragraph of page 8, the names of some of the clusters change (I believe based on which hemisphere they are in). for example, Cluster 4 is referred to as the language network in one sentence and then the right default mode network in the next. Perhaps they could more clearly explain the naming convention up front.

Reply

We thank the reviewer for this comment. In some paragraphs, the networks contained in Cluster 4 were abbreviated, and now we had unified the description of Cluster 4 as “DMN and language network” throughout the MS.

2) the authors do not mention that they control for multiple comparisons for any of the correlations in the Results section. That important detail is buried in the Methods. Furthermore, I am not sure whether they properly corrected for the multiple comparisons between the 4 clusters and 13 cognitive tests mentioned in the Results because the number of cognitive tests is not mentioned in the Methods.

Reply

Multiple comparison correction was performed across all brain measures used in our paper, with 29 comparisons (4 MLI + 4 LF + 4 LR + 10 DLI correlation + 3 dwelling time +3 fraction + transition = 29), see page 18 in our previous submission. We thank the reviewer for pointing this out and we have clarified this multiple comparison correction in page 7 and page 8.

3) The authors do not report the p-values for the correlations in the last paragraph of page 8.

Reply

We added p-values for all these correlations in the revised manuscript, see page 9.

Finally, we would like to thank the 2^st^ reviewer again especially for the insightful comments and suggestions regarding the stability of the results and influence of head movements, and also the referring to prior work in the field, all have helped us greatly in improving our MS.

**Reviewer #3**: Large scale studies on resting state functional connectivity (RSFC) have identified time-varying functional lateralization as one hallmark of endogenous brain activity fluctuations (e.g. Liu et al., PNAS 2009; Wang, Buckner and Liu, J Neurosci 2014) and related these fluctuations with task-related activity to elucidate their functional significance. These studies have provided evidence that functional lateralization is rather flexible than stable over time although lesion studies indicate very consistent hemispheric specializations. The relation between lesion effects and the more dynamic network changes is still enigmatic.

In their submitted work, Wu and colleagues now analyze RSFC from the Human Connectome Project and claim to have revealed brain laterality dynamics that support human cognition. They found stronger and more consistent correlations between regions belonging to the left language and default mode network and the global signal of the left hemisphere compared to the relation between the right homotopes and the right hemisphere's global signal. Right-lateralized "global" intra-hemispheric RSFC was primarily found for the right frontoparietal network and the visual cortex, for which variability was rather high. The authors found negative correlations between the language and default mode network lateralization and the lateralization of other resting state functional networks, define the dwelling time in such states during rest and investigated the relationship between functional laterality, structural connectivity and even heritability. Finally, they try to link their observations with behavior by performing multiple correlations with various off-line behavioral scores. The analyses are elaborate and in part convincing. In the following, I will try to highlight strengths and weaknesses of the current draft and provide some suggestions that may help improve the paper.

Reply

We thank the 3^rd^ reviewer very much for the comments that “the analyses are elaborate and in part convincing”. We also want to thank the reviewer for providing us with a detailed, retrospective view in the field of laterality, which helps to put our work into position, and we have involved the relevant literatures mentioned by the reviewer in the revised MS (page 3, page 12-13).

First of all, the approach to study functional lateralization over time is very interesting and follows the work by Randy Buckner. The way the authors approach functional lateralization, however, is too narrow to provide a comprehensive picture. "Laterality" is defined here as stronger RSFC between a region of interest (ROIs defined based on the Glasser parcellation scheme, Nature 2016) and the global, averaged time course of all other ipsilateral ROIs compared to the homologue RSFC in the other hemisphere. Although at least one previous study has used this approach (McAvoy et al., Cerebral Cortex 2016), this definition of laterality could be criticized for the basic problems of averaging and also restricts the search space to intra-hemispheric connectivity that is strong enough to influence the hemisphere's global signal. This likely is a too coarse measure to detect network interactions underlying human cognition and completely neglects interhemispheric interactions. It seems counterintuitive to use an elaborate parcellation scheme only to study correlations with averaged time courses in the end. I appreciate the effort the authors made to demonstrate that analyses of hemispheric differences in intra-hemispheric ROI to ROI interactions would reveal a comparable pattern (partly arguing against averaging problems), but, still, this does not tell us anything about interhemispheric interactions (be they homotopic or heterotopic) or specific intra-hemispheric connections that contribute to human cognition. Please see, amongst others, Wang et al., J Neurosci 2014, for a comprehensive picture of the role of intra- and interhemispheric interactions in RSFC and their functional significance.

Moreover, laterality indices, as the one used by the authors, do not reveal whether observed functional lateralization results from a shift in interhemispheric balance or whether the effect is locally generated either in the left (e.g. increased RSFC) or the right hemisphere (e.g. decreased RSFC). I suggest adding this information by providing the raw RSFC values, because this information is critical for the interpretation of effects (a decrease in intra-hemispheric global connectivity of right homologues of language regions likely has different meaning compared to increased intra-hemispheric global connectivity of left language regions). This would also help in better understanding the laterality reversals that the authors interpret as excessive fluctuations in functional lateralization.

Reply

We thank the reviewer very much for pointing out the potential problem with our approach, which we have fully examined and carried all our necessary investigations to improve our work. While our global-signal based laterality index has several advantages like being computationally convenient and is highly correlated to traditional laterality index, it also has some disadvantages like cannot detect specific ROI-to-ROI interactions underlying dynamic laterality. We have thus carried out further analysis to examine which functional subnetworks may affect DLI in the identified 4 clusters, as a remedy to the disadvantage of our DLI approach. **In the revised MS, we have also tuned down our description when it comes to its advantage (page 5) and pointed out its disadvantage in the limitation part (page 14), so that the comparisons to other approaches would be fair.**

We have made the following revisions and investigations:

First, we embedded the relevant literatures mentioned by the reviewer in the introduction part and provided a more comprehensive picture of laterality, see page 3, page 12-13 in the MS.

Second, we agreed with the reviewer that the laterality index using global signal may have the problem of averaging and might be too coarse to detect network interactions underlying cognition, which we have pointed out in the Limitation section. In response to the problem raised by the reviewer, we have performed extensive further analysis as below:

To identify which functional connections may play important roles in the dynamic change of laterality, we adopted a regression-based approach to investigate which sub-networks (we used 9 major networks of all 12 networks in Cole-Anticevic Brain-wide Network Partition with 3 minor network being excluded, following Ito et al.^17^ The 3 minor networks are posterior-multimodal, ventral-multimodal and orbito-affective, and only had very few regions and could not be integrated into the major networks) may affect the dynamic laterality of the 4 identified clusters. Specifically, we tested whether the linear regression coefficients of network-averaged functional connectivity on the DLI time series of the four clusters were significantly greater or less than 0 (one-sample t test, FDR q<0.05). More details can be found in Supplemental Methods.

We found that higher level of lateralization of a cluster, either right-lateralized (for Cluster 1-3) or left-lateralized (Cluster 4) was associated mainly with weaker functional connectivity of the cluster with the contralateral high-order networks (e.g., FPN and DMN). Stronger functional connectivity of the cluster with its ipsilateral high-order networks also plays a role but has less contribution. In terms of homotopic/non-homotopic connectivity, higher level of lateralization of a cluster is associated more with decreased non-homotopic connections (compared to homotopic connections). In general, dynamic changes of functional connections associated with higher-order brain regions play an important role in the dynamic laterality, see Supplemental **Figure 12**. We have briefly mentioned these results in the MS (page 8).

*Supplemental Figure 12. The influence of dynamic functional networks on the DLI time series of four clusters. We constructed the dynamic network matrices of 9 major networks (Cole-Anticevic Brain-wide Network Partition) of bilateral hemispheres, and then calculated their regression coefficients on DLI for each subject in each run. We averaged the regression coefficients of the 4 runs of each subject and tested whether they were significantly greater than or less than 0 with single-sample t test. The figure shows the t-values and only FDR corrected brain regions are shown. The connections within the black box are homotopic network connections. Vis1, Visual1; vis2, Visual2; smn, Somatomotor; con, Cingulo-Opercular network; dan, Dorsal-Attention network; lan, Language network; fpn, Frontoparietal network; aud, Auditory network; dmn, Default Mode network.*

Finally, are some explanations why we used global-signal based laterality index: In HCP data, there are about 1000 subjects. As the generally used laterality index is based on FC among ROIs, given the HCP-MMP parcellation with 360 ROIs we adopted, this leads to 1000*360*359/2 FCs. Considering also the high temporal resolution of this data (about 1200*4 time points each) in calculating the FC, all together these will cause heavy computational burden. Our initial analysis did adopt the traditional ROIs-based laterality index ^18,19^. Because this turns out to be too time-consuming, we make a compromise and used the laterality index based on the global signal, which is about 180 times faster than the traditional laterality index. To verify this global-signal based laterality index, we have shown in our previous submission that GS-based index is indeed highly correlated with the ROI based laterality index both numerically and mathematically, see Supplemental Methods and Supplemental Fig. 1.

How can the authors assure that the correlation coefficients between regional time courses with the hemispheres' global signal indeed reflects functional connectivity between regions and not only a stronger contribution of one resting state functional networks activity to the global hemispheric time course? I believe this point is critical for their interpretation.

Reply

While our global-signal based laterality index has several advantages like computationally convenient and is highly correlated to traditional laterality index, it also has some disadvantages like cannot detect specific ROI-to-ROI interactions underlying dynamic laterality. In the revised MS, we have pointed out the disadvantages in the limitation part (page 14). To solve this problem, we have made further analysis (Please see also our response to the above problem) to examine which functional network would affect DLI.

.

When reading previous papers on RSFC, the comparison between RSFC and task-related activity in the same subjects was of great help to understand the significance of findings for cognition. In the submitted work, the authors chose to generate a link between brain and behavior by performing numerous correlations of "brain laterality dynamics" with various off-line behavioral scores that likely reflect a mixture of trait- and offline state-related effects. Besides the (in my opinion) insufficient statistical correction for the number of tested correlations, this approach is hard to justify. How should dynamic laterality measured during four measurements of resting state mechanistically relate to behavioral measures that were acquired off-line? Indeed, it seems that the used correlation between local time series and the hemisphere's averaged time course is more robust than the autonomy index (see page 5). This suggests that the used laterality index likely does not necessarily reflect dynamics that relate to cognitive states. To my knowledge, the HCP included functional imaging. Don't the authors see the chance to relate their findings with task-related activity instead of off-line behavioral scores to back up their claims (cw., e.g. Wang et al., J Neurosci 2014 and Liu et al., PNAS 2009)? In my opinion, the current version of the manuscript unfortunately falls behind previous reports on the relationship between RSFC and behavior.

Reply

We thank the reviewer for pointing out this important problem. In our previous submission, we have carried out extensive calculations to demonstrate that the laterality index obtained by traditional ROI-based approach and global-signal approach are indeed highly correlated (Supplemental Fig. 1).

As suggested by the reviewer, we analyzed the task-state fMRI, we extracted the BOLD time series from HCP language task and calculated the DLI using the same sliding-window approach (across the whole run) as in resting-state (please also see the next page why HCP language task is not ideal for dynamic laterality analysis). We found:

(1) Task-state LF (laterality fluctuation) and LR (laterality reversal) generally showed similar patterns with the resting state (Figure 1 below). the task-state and resting-state DLI maintained some similarity in the spatial distribution (for example, high order regions maintained the highest mean laterality and the lowest laterality reversal, and the visual areas showed the largest LF).
(2) The mean of DLI of the left and right hemispheres became more prominent (Figure below, A): left lateralization was observed in all regions of the left hemisphere, and right lateralization was observed in all regions of the right hemisphere, where the regions associated with language processing, such as IFG, STG and TPJ, showed the highest level of lateralization, which are involved in language processing and social function in previous studies^20,21^.
(3) For temporal clustering analysis, we identified 3 states. The fraction of state 2 showed a negative correlation with the accuracy of language task (r=0.3, p=0.002) and the transform of the different states showed a positive correlation with the language task response time (r=-0.247, p=0.013) at uncorrected p<0.05 level.

(4) Since the spatial clustering results for task-fMRI are very unstable (we repeated 1000 times of clustering analysis, among which 20.9% obtained the optimal number of classes is 3 and 79.1% obtained the optimal number of classes is 4 and 1000 times, the average Dice coefficient between different classification schemes is 0.29±0.21, and the spatial overlap ratio is relatively low), therefore we used clustering results (4 clusters) from resting state data. We found that cluster-level MLI, LF, and LR did not show significant correlations with language, CardSort, and ProcSpeed (Figure below, E; FDR q=0.05).

*(A-D) task -DLI results. (A) mean laterality index (MLI). (B) laterality fluctuation (LF). (C) laterality reversal (LR). (D) the results of timing clustering. (E) correlation between cognitive measures and indicator of dynamic lateralization. The cross indicates FDR q<0.05. C1-C4: correlation between dynamic laterality index of Cluster 1 and Cluster 4, and so on.*

**There are several important reasons why significant correlation between task state DLI and cognitive performance was not observed:**

(a) HCP language task is not a pure language task, and each run includes a story comprehension condition (4 trials) and a mathematical computation condition (9 trial, each trial is about 12~13s). The two conditions are presented alternately, **therefore the laterality changes are contaminated by the mathematical computation trails and that the correlation between dynamic laterality and language performance would be vague.**

(b) If we extract the language-related trails, the corresponding BOLD signals would be too short (about 20~30s, less than a time window length) to carry out a sliding-window analysis. Thus, in the above analysis (1-4), we used a sliding window approach across the entire run, although this has a problem of mixed language and mathematical trials.

**In summary, the HCP task-fMRI may not be ideal for dynamic laterality analysis.** It will be our future work to find a suitable language task-fMRI data for dynamic laterality analysis (for example, reading a long article in scanner) and for investigating the relationship between DLI and language processing.

We have cited the literatures provided by the reviewer (Wang et al., J Neurosci 2014 and Liu et al., PNAS 2009, see page 3 and page 12-13) to introduce background about asymmetry and verify the consistency of our results with previous authoritative results.

I particularly liked the explored correlations between time courses of the spatial clusters. Laterality time courses of cluster 4 are negatively correlated with the time courses of the remaining clusters which suggests indeed a special role for the language and default mode network. However, the dissociation between the two (language and DMN) could have been explored further. Do they lateralize conjointly? Could this effect be driven by their relative contribution to the global signal compared to the other clusters? What would be the prediction for the functional lateralization during an active language task for both networks?

Reply

We thank the reviewer for the positive comments of the spatial clustering analysis being a key part of the dynamic laterality analysis. For the question "if language and DMN lateralize conjointly”, we believe that this is the case. Across all time windows, the laterality indices for language network and DMN are positively correlated at 86.2% participants, indicating that when one cluster is left-lateralized, the other is also left-lateralized.

We examine the relationship between the contribution of the language network and DMN network to GS (measured by the regression coefficient) and the DLI covariance between DMN and language network. We find that they do have significant positive correlations (DMN contribution & DMN-LAN covariance: r=0.23, p=7e-13; LAN contribution & DMN-LAN covariance: r=0.069, p=0.03).This may indicate that global signals have some influence on DLI, so we repeated our main analyses using BOLD signals with GS regressed out and found that the results did not change much (see Supplemental Figure 10).

*Supplemental Figure 10. The result with global signal regressed out. (A) the effect of global signal to DLI (t value of regression coefficient). (B) mean laterality index (MLI). (C) laterality fluctuation (LF). (D) laterality reversal (LR). (E) result of spatial clustering. (F) the results of timing clustering. (G) correlation between cognitive measures and indicator of dynamic lateralization. The cross indicates FDR q<0.05. C1-C4: correlation between dynamic laterality index of Cluster 1 and Cluster 4, and so on.*

We calculated the correlation between DLI time course of DMN and language network and found that they are also positively correlated. In task-state fMRI, about 70% of subjects showed a positive correlation between the language network and DMN (r=0.022±0.038), indicating that these two networks remained co-lateralized both at rest and in the task state. We hope the reviewer could let us know if we mis-understand this comment.

Minor points: For lateralization analyses, symmetrical templates are used for normalization to account for structural differences between the hemispheres (see e.g. Karolis et al., Nature Comm 2019). It seems that this has not been done here. Please specify. Could you please argue why in the case of parcellated ROIs this should not be critical?

Reply

We thank the reviewer for this suggestion. We chose the parcellated ROIs instead of symmetric template mainly based on the following reasons:

(1) for dynamic fMRI analysis, vertex-level analysis would be too time-consuming and a fine-partitioned symmetric parcellation can effectively reduce the computational load. The HCP-MMP parcellation contains 180 pairs of homotopic regions from two hemispheres which we believe can meet our needs.

(2) Parcellation-based methods were commonly adopted in previous asymmetry studies. For example, several large-sample studies from ENIGMA used parcellation (containing 34 pairs of homotopic regions) rather than symmetric template to calculate brain asymmetry ^22-25^.

(3) The laterality measure we adopted does not directly compared two homotopic regions (in terms of their imaging indicators), and each brain region was assigned a laterality index. Therefore, two homotopic regions do not need to be identical in terms of constituent voxels. Considering that the HCP-MMP parcellation contains 180 pairs of homotopic regions, the potential influence from template would presumably be small.

The Figure 3a suggests the cluster 3 to be stronger in the left and cluster 4 in the right hemisphere. The text (brackets on page 6) suggests the contrary. Please correct.

Reply

We checked our Figure 3a and found our figure might be misleading: Cluster 1 was placed at the bottom and Cluster 4 was placed at the top. We have changed our figure to make it clearer. We thank the reviewer for such a careful check of our results.

From the text it does not become clear that probabilistic fiber tracking has been performed in different subjects (100 unrelated subjects). Please mention this appropriately also outside the methods section.

Reply

We thank the reviewer for this advice. We have added the relevant statement at Results section (page 9) as “We used diffusion imaging data from a subset of HCP S1200 (HCP Unrelated 100) to constructed two kinds of structural connection matrix, fractional anisotropy (FA) matrix and fiber number (FN) matrix for each subject”.

The title is misleading, because the link between laterality dynamics and human cognition is only indirectly studied here.

Reply

We agree with the reviewer that the title may be misleading and have revised it as “Dynamic architecture of brain lateralization at rest”.

On page 11, the authors state "these results therefore indicate optimal cognitive performance requires a dynamic balance between interhemispheric information exchange and intra-hemispheric information processing." This clearly cannot be claimed based on the presented analyses results. Some parts of the discussion are quite speculative, particularly when the weak correlations with behavioral measures are discussed.

Reply

We thank the reviewer for pointing this out. We have revised the statement in page 12 as "these results therefore indicate optimal cognitive performance may be related to a dynamic balance between interhemispheric information exchange and intra-hemispheric information processing" to tune down some of our claims.

The authors state in the manuscript that the data are deidentified, but in the submission form they argue against data sharing, because "use is restricted for researchers who meet the criteria for access to confidential data". There seems to be a misunderstanding there.

Reply

Our description is not clear enough that leads to misunderstanding: HCP imaging data and most behavioral data are publicly available, but subjects' personal information or “restricted data” (for example, information about zygosity and parents) is not, and needs application. We have thus revised these statements in Data Availability (page 21) to make it clearer.

Finally, we would like to thank the 3^st^ reviewer again for highlighting the strengths of our work and pointing out potential weaknesses of the adopted laterality measures. The suggestions provided by the reviewer have helped us significantly in improving our MS.

**Reference**

1.	Allen, E.A.*, et al.* Tracking Whole-Brain Connectivity Dynamics in the Resting State. *Cerebral cortex* **24**, 663-676 (2012).

2.	Douw, L., Wakeman, D.G., Tanaka, N., Liu, H. & Stufflebeam, S.M. State-dependent variability of dynamic functional connectivity between frontoparietal and default networks relates to cognitive flexibility. *Neuroscience* **339**, 12-21 (2016).

3.	Falahpour, M.*, et al.* Underconnected, but not broken? Dynamic functional connectivity MRI shows underconnectivity in autism is linked to increased intra-individual variability across time. *Brain connectivity* **6**, 403-414 (2016).

4.	Li, R.*, et al.* Differential patterns of dynamic functional connectivity variability of striato–cortical circuitry in children with benign epilepsy with centrotemporal spikes. *Human brain mapping* **39**, 1207-1217 (2018).

5.	Bassett, D.S.*, et al.* Task-based core-periphery organization of human brain dynamics. *PLoS computational biology* **9**, e1003171 (2013).

6.	Braun, U.*, et al.* Dynamic brain network reconfiguration as a potential schizophrenia genetic risk mechanism modulated by NMDA receptor function. *Proceedings of the National Academy of Sciences* **113**, 12568-12573 (2016).

7.	Gonzalez-Castillo, J.*, et al.* Tracking ongoing cognition in individuals using brief, whole-brain functional connectivity patterns. *Proceedings of the National Academy of Sciences* **112**, 8762-8767 (2015).

8.	Zang, Y.F.*, et al.* Altered baseline brain activity in children with ADHD revealed by resting-state functional MRI. *Brain & Development* **29**, 83-91 (2007).

9.	Dong, M.*, et al.* Altered baseline brain activity in experts measured by amplitude of low frequency fluctuations (ALFF): a resting state fMRI study using expertise model of acupuncturists. *Frontiers in Human Neuroscience* **9**, 99 (2015).

10.	Gee, D.G.*, et al.* Low frequency fluctuations reveal integrated and segregated processing among the cerebral hemispheres. *Neuroimage* **54**, 517-527 (2011).

11.	Yeo, B.T., Krienen, F.M., Chee, M.W. & Buckner, R.L. Estimates of segregation and overlap of functional connectivity networks in the human cerebral cortex. *Neuroimage* **88**, 212-227 (2014).

12.	Shine, J.M. Neuromodulatory influences on integration and segregation in the brain. *Trends in cognitive sciences* (2019).

13.	Li, J.*, et al.* Topography and behavioral relevance of the global signal in the human brain. *Scientific reports* **9**, 1-10 (2019).

14.	Laumann, T.O.*, et al.* On the stability of BOLD fMRI correlations. *Cerebral cortex* **27**, 4719-4732 (2017).

15.	Friston, K.J., Williams, S., Howard, R., Frackowiak, R.S.J. & Turner, R. Movement‐related effects in fMRI time‐series. *Magnetic resonance in medicine* **35**, 346-355 (1996).

16.	Yan, C.-G., Wang, X.-D., Zuo, X.-N. & Zang, Y.-F. DPABI: data processing & analysis for (resting-state) brain imaging. *Neuroinformatics* **14**, 339-351 (2016).

17.	Ito, T.*, et al.* Cognitive task information is transferred between brain regions via resting-state network topology. *Nature communications* **8**, 1-14 (2017).

18.	Liu, H., Stufflebeam, S.M., Sepulcre, J., Hedden, T. & Buckner, R.L. Evidence from intrinsic activity that asymmetry of the human brain is controlled by multiple factors. *Proceedings of the National Academy of Sciences of the United States of America* **106**, 20499-20503 (2009).

19.	Wang, D., Buckner, R.L. & Liu, H. Cerebellar asymmetry and its relation to cerebral asymmetry estimated by intrinsic functional connectivity. *Journal of Neurophysiology* **109**, 46-57 (2013).

20.	Friederici, A.D. *Language in our brain: The origins of a uniquely human capacity*, (MIT Press, 2017).

21.	Adolphs, R. The social brain: neural basis of social knowledge. *Annual review of psychology* **60**, 693-716 (2009).

22.	Kong, X.-Z.*, et al.* Mapping cortical brain asymmetry in 17,141 healthy individuals worldwide via the ENIGMA Consortium. *Proceedings of the National Academy of Sciences* **115**, E5154-E5163 (2018).

23.	Cao, Z.*, et al.* Mapping cortical and subcortical asymmetries in substance dependence: Findings from the ENIGMA Addiction Working Group. *Addiction Biology*, e13010 (2021).

24.	Postema, M.C.*, et al.* Altered structural brain asymmetry in autism spectrum disorder in a study of 54 datasets. *Nature communications* **10**, 4958 (2019).

25.	Kong, X.-Z.*, et al.* Mapping brain asymmetry in health and disease through the ENIGMA consortium. (2019).
