# Supplementary material for: Dynamic changes in brain lateralization correlate with human cognitive performance
Source: PLoS Biol. 2022 Mar 17;20(3):e3001560. doi: 10.1371/journal.pbio.3001560 (PMC8929635; doi:10.1371/journal.pbio.3001560)
Supplement: Attachment — Submitted filename: Response to Editors 1221_DV_Comments.docx [file pbio.3001560.s027.docx]

Dear Editor,

First of all, we would like to thank you for the rapid processing of our manuscript. We are encouraged that the first two reviewers are satisfied with the revised manuscript. We also thank the 3^rd^ reviewer for the additional suggestions which allowed us to further improve our manuscript. The further revisions we have made are marked in red in the revised manuscript. Below is a point-by-point response to the reviewer’s comments.

REVIEWS:

Reviewer #1: I think the authors have done a nice job addressing previous reviewers comments. I have no further comments.

Reviewer #2: The authors have significantly improved upon the original submission. I am satisfied with the revised manuscript and the authors' thorough replies to my concerns.

Reviewer #3: The authors now report a considerable number of additional analyses that attempt to deal with the reviewers' comments. Unfortunately, the changes made to the manuscript were only very limited. The authors chose to bury the new analyses in the supplementary material, that now encompasses 13 Figures and the old tables. The text in the supplementary material is very preliminary, contains many orthographic mistakes and does not convey sufficient specific information on the added analyses. Also, the supplementary text and figures appear unstructured.

Response: We thank the reviewer for providing further suggestions which we have considerably improved out manuscript. In our revision we have re-structured and modified the supplementary materials, corrected language mistakes and moved some of the key supplementary results into the main manuscript. Note: The page number used in this file is for the **Revised Article with Changes Highlighted**.

The most interesting aspect that arose from the revision was the new result that functional lateralization was more strongly related to diminished interhemispheric connectivity rather than increased intra-hemispheric connectivity over time.

Response: We thank the reviewer for appreciating our additional analysis on the possible factors that influence dynamic functional laterality. We have added a long paragraph in the **Results** section to highlight these findings (with a figure, see page 8) and provide relevant discussion in the revised manuscript (see page 14). We now mention this result briefly in the Abstract, “the laterality dynamics are associated with functional connectivity changes within higher-order brain networks”.

I list only the most important points I see that were insufficiently addressed.

The control analyses are not sufficiently described. To name only a single point: How exactly did the authors regress the global signal out when one hemisphere's global signal was the critical time course for estimating functional connectivity with individual ROIs? Shouldn't this result in quasi zero correlation? Or did the authors regress out the average global signal over both hemispheres in their control analysis? I will let reviewer 2 comment on the movement issue; I am not entirely sure whether the authors convincingly dealt with her/his comment.

Response: We apologize for the lack of clarify in our explanation of the procedures employed in the above-mentioned analysis. Indeed, we regressed out the global signal **of the whole brain** (GS), which resulted in the sum of the left (GS_L_) and the right hemisphere global signal (GS_R_) being 0, i.e., GS_L_+GS_R_=0, rather than GS_L_=0, or GS_R_=0. When the global signal of the left hemisphere (GS_L_) is positive, the global signal of the right hemisphere (GS_R_) is equal to the negative number of the GS_L_, so the correlation coefficients can still be calculated between hemispheric global signal and individual regions (which may not be 0). We have added additional notes to the **Result** section (page 11) in the revised manuscript to clarify this point.

The authors still do not correct sufficiently for the number of tested correlations (neither the numbers of off-line behavioral measures seem to be considered, nor the number of the various correlation analyses in the different results subsections).

Response: We thank the reviewer for raising this important point. We now clarify that the number of various correlation analyses in different **Results** subsections (4 MLI + 4 LF +4 LR+10 LCV + 3 dwelling time + 3 fraction + 1 transition = 29) were in fact considered in the main analysis. Please see page 7 (“Multiple comparisons were performed using the False Discovery Rates (FDR) method for all laterality measures used in the analyses”) and page 8 (“Multiple comparisons were performed using the FDR method for all laterality measures used in the analyses (multiply comparison times=4 MLI + 4 LF + 4 LR + 10 DLI correlation + 3 dwelling time +3 fraction + transition = 29”).

We agree with the reviewer that the correction for the number of off-line behavioral measures is valuable. As such we conducted additional multiple comparison correction in accordance with the reviewer’s suggestion (FDR q=0.05/13=0.0038) and all that results passed this conservative FDR correction were highlighted in the main text (for example, “r=…, FDR q<0.05/13”). Overall, the main results remained the same: higher cognitive flexibility (CardSort) was associated with more right-lateral Cluster 3, more left-lateral Cluster 4, higher whole-brain LF, and lower LR in Cluster 3/4. Higher processing speed (ProcSpeed) was associated with lower LR of cluster 3. Better performance to understand stories (LanDiff) was associated with stronger LF and lower LR in Cluster 3 (Supplemental Table 1-3). Corresponding revisions were made in pages 7-8.

The authors did not change the speculative discussion on the relation between their findings in resting state and cognition. Mentioning the limitation of the study is not enough, when unjustified claims are made in the discussion. I appreciate the effort the authors made by analyzing task-related activity. Unfortunately, the authors did not find a relationship and argue in the rebuttal letter, why this could be the case. However, the only information I found on this issue in the revision, was the task description in the supplementary material. To improve the discussion, I suggest removing the speculations on cognition and rather focus on the claims the authors can make based on resting state data.

Response: We appreciate the reviewer’s comments on this point, and we have now revised the discussion section accordingly. Specifically, we removed any speculative explanations on language ability and focused more on interpretations based on resting state data (page 12). We have also revised the abstract and used “cognitive abilities” instead of “language ability and cognitive flexibility”.

In addition, we also highlighted that using resting-state fMRI data to predict off-line behavioral measures are widely accepted in previous studies (for example, resting state human functional connectome was used in predicting attention task performance, personality traits and intelligence [1-3]) which collectively suggest a trait level relationship between brain and behavior. In line with this, our main results (CardSort/LanDiff are associated with greater LF and smaller LR) also survived under stricter multiple comparison correction (FDR q<0.05/13), therefore we reserved part of the discussion about the relationship between the laterality of resting state and intelligence.

Although the authors added some references to previous studies investigating lateralization of resting state dynamics, they did not put their findings into context. What do we learn from this new report compared to the previous ones? What did the old reports suggest that was disproven by the new data? Which aspects were replicated?

Response: We thank the reviewer for their assessment. We believe that our results extend the traditional static laterality analysis to its dynamic counterpart. To further highlight this, we now provide some discussion points on the commonalities and differences between our research and prior studies in the revised manuscript (pages 11-12). For example, we state that “This study proposed a framework for dynamic laterality analysis. The time-averaged dynamic laterality index well replicated previous findings in terms of the basic (static) spatial patterns of functional lateralization, while the time-varying laterality measures provided new insights into the dynamic changes of lateralization in resting-state. The current study revealed the dynamic nature of laterality across the brain and its cognitive significance.” “…This suggested laterality in lower-order regions is more flexible, consistent with previous research about intrinsic time scale of higher and lower-order regions. That is, the bilateral sensory areas need to process constantly changing sensory inputs and integrate them with information from the contralateral hemisphere in real time to form accurate and coherent perception.”

The authors did not explore well enough what their lateralization measure actually reflects. For example, the measure would not pick up a change in correlation between a left ROI and five right ROIs in case it is accompanied by a change in correlation between the same left ROI and five other right ROIs of the same magnitude. Because the choice of the lateralization measure is critical for the interpretation, I would at least have expected a clear statement what a correlation of a local signal with a global signal means, especially when the global signal of the ipsilateral hemisphere is "contaminated" more strongly by the ROIs activity that the other hemisphere's global signal.

Response: We thank the reviewer for raising this important issue. Following the reviewer’s suggestion, we further explained the indices of laterality in the **Results** section and made a clear statement about what a correlation of a local signal with a global signal represents: “GS-based laterality index of a ROI measures whether the activity of the ROI is more synchronized with the left hemisphere or the right hemisphere. For an ROI, its correlation with GS of left hemisphere (GS_L_) represents its synchronization with all regions of the left hemisphere, and its correlation with the right hemisphere’s GS (GS_R_) represents the synchronization with the overall right hemisphere. Higher correlation of a ROI with GS_L_ compared to GS_R_ suggests left lateralization and vice versa.” (page 4).

The reviewer indicates that our method cannot reflect which functional connections dominate the laterality changes of ROI *A*, which may come from *A-B* connections or *A-C* connections. We agree with the reviewer that our method put more emphasis on the overall synchronization of ROI *A* with both hemispheres and less on specific brain regions, so we adopted a compensatory approach to try to solve this problem (see **Method** section, page 19). We focused on investigating which functional connections changed most when the laterality of ROI *A* changes, that is, the patterns of association between the laterality time series of ROI *A* and each functional connectivity time series, so as to confirm which connectivity may be related to the laterality changes.

Because the ROIs within a cluster display a similar trend of laterality changes over time, we obtained dynamic laterality of the 4 clusters by averaging the laterality time series of ROIs within each cluster. We then averaged functional connectivity within/between the 24 subnetworks, therefore there are 24*23/2=276 subnetwork-level functional connections. Specifically, we built linear regression models of cluster-level DLI time series on sub-network level dynamic functional connectivity (DFC) and performed one-sample t test on the regression coefficient (β), to investigate whether the influence of DFC on DLI was significantly greater than 0 (positive coupling) or less than 0 (negative coupling) (page 9&19, Fig. 5a)

*Figure 5. Association between dynamic laterality time series of four clusters and dynamic functional connectivity within/between 24 subnetworks. (a) Analysis pipeline. We established linear regression models of cluster-level DLI time series on sub-network level dynamic functional connectivity (DFC) and performed one-sample t-test on the regression coefficient (β) to investigate whether the influence of DFC on DLI was significantly greater than 0 (positive coupling) or less than 0 (negative coupling). (b) the association between dynamic laterality time series of Cluster 1/2/3/4 and the DFC within/between 24 subnetworks (according to Cole-Anticevic Brain-wide Network Partition). Only functional connections with highly significant t value of regression coefficient (with p<1e-200* *or |t|>40, one-sample t-test) is being shown since there are too many significant connections. Here positive laterality index indicates left-lateralization. Colors of points indicate the network arrangement of the Cole-Anticevic Brain-wide Network Partition (CAB-NP). Vis1, Visual1; vis2, Visual2; smn, Somatomotor; con, Cingulo-Opercular network; dan, Dorsal-Attention network; lan, Language network; fpn, Frontoparietal network; aud, Auditory network; dmn, Default Mode network; pmm, Posterior-Multimodal; vmm, Ventral-Multimodal; ora, Orbito-Affective. L, left hemisphere; R, right hemisphere.*

Our results demonstrated the importance of high-order networks (DMN/FPN/language network, main covered by our Cluster 4) in the laterality changes of the four clusters: the increased level of right-lateralization of Cluster 1/2/3 was associated with their decreased functional connectivity with Cluster 4 on the left hemisphere. Similarly, the increased level of left-lateralization of Cluster 4 was related to its decreased functional connectivity with Cluster 1/2/3 at the right hemisphere, suggesting that Cluster 4 might be critical for the lateralization state of the whole brain. This result could answer the questions raised by the reviewer: when a right ROI becomes more right-lateralized, changes occur in its functional connectivity between this ROI and five left higher-order ROIs rather than five other left sensorimotor ROIs of the same magnitude. Related discussion can be found on page 14.

The reviewer noted that the hemispheric global signal may be contaminated by the ipsilateral ROI. We agree with the reviewer that including an ipsilateral ROI in calculating its correlation with ipsilateral global signal might slightly increase the correlation coefficient. However, we also believe that the effect of this increased correlation on laterality (distribution of dynamic laterality statistics across brain, or brain-behavior correlation) would be weak: previous studies have shown that BOLD time series of homotopic regions are highly positively correlated (close to 1) [4]. Therefore, when comparing the correlation between a left ROI and the left global signal, and its correlation with right global signal, the correlation coefficient between the ROI and itself is 1, while the correlation coefficient between the ROI and its right homotopic connection is also close to 1, which cancels out much of the "contaminated effect" on the left. In addition, to completely exclude possible contamination, we re-constructed GS' by removing the ROI *A* from its ipsilateral GS and repeated our analysis. We found no major changes in the results, indicating that the contaminated effect may have only trivial influence on the laterality index (page 11, Supplemental Result & Supplemental Fig. 15).

*Supplementary Figure 15. (A-F) The influence of removing the ROI from its ipsilateral global signal in calculating the laterality index. That is, we removed the ROI A from its ipsilateral global signal. (A) mean laterality index (MLI). (B) laterality fluctuation (LF). (C) laterality reversal (LR). (D) result of spatial clustering. (E) the results of temporal clustering. (F) correlation between cognitive measures and indicator of dynamic lateralization. The cross indicates FDR q<0.05. C1-C4: correlation between dynamic laterality index of Cluster 1 and Cluster 4, and so on.*

In sum, the expectation of a substantially revised manuscript that I got when reading the rebuttal letter was unfortunately not met.

Response: We would like to thank reviewer #3 again for their suggestions. We have carefully revised the whole manuscript to clarify any vague points and imprecise descriptions and inappropriate discussions. While our laterality measurement may have some advantages in computational efficiency, it lacks spatial resolution and thus we have provided extensive post-hoc analysis to identify which subnetworks may have an important role in affecting the laterality changes. We hope that the revised manuscript will now meet the reviewer’s expectations.

**Reference**

1.	Shen X, Finn ES, Scheinost D, Rosenberg MD, Chun MM, Papademetris X, et al. Using connectome-based predictive modeling to predict individual behavior from brain connectivity. nature protocols. 2017;12(3):506-18.

2.	Dubois J, Galdi P, Paul LK, Adolphs R. A distributed brain network predicts general intelligence from resting-state human neuroimaging data. Biological Sciences. 2018;373(1756).

3.	Dubois J, Galdi P, Han Y, Paul LK, Adolphs R. Resting-state functional brain connectivity best predicts the personality dimension of openness to experience. Personality neuroscience. 2018;1.

4.	Zuo XN, Kelly C, Di Martino A, Mennes M, Margulies DS, Bangaru S, et al. Growing Together and Growing Apart: Regional and Sex Differences in the Lifespan Developmental Trajectories of Functional Homotopy. Journal of Neuroscience. 2010;30(45):15034-43. doi: 10.1523/jneurosci.2612-10.2010.
